# Single-cell RNA sequencing reveals peripheral blood leukocyte responses to spinal cord injury in mice with humanised immune systems

**DOI:** 10.1186/s12974-024-03048-0

**Published:** 2024-03-01

**Authors:** Ellen R. Gillespie, Laura F. Grice, Isabel G. Courtney, Hong Wa Lao, Woncheol Jung, Sonny Ramkomuth, Jacky Xie, David A. Brown, James Walsham, Kristen J. Radford, Quan H. Nguyen, Marc J. Ruitenberg

**Affiliations:** 1 School of Biomedical Sciences, Faculty of Medicine, The University of Queensland, Brisbane, QLD 4072 Australia; 2 Institute for Molecular Bioscience, The University of Queensland, Brisbane, Australia; 3 Neuroinflammation Research Group, Centre for Immunology and Allergy Research, Westmead Institute for Medical Research, Sydney, Australia; 4 Intensive Care Unit, Princess Alexandra Hospital, Brisbane, Australia; 5 Mater Research Institute, The University of Queensland, Translational Research Institute, Brisbane, Australia; 6 Centre for Immunology and Allergy Research, Westmead Institute for Medical Research, The University of Sydney, Sydney, Australia; 7 Institute for Clinical Pathology, New South Wales Health Pathology, Sydney, Australia; 8 Medical School, Faculty of Medicine, The University of Queensland, Brisbane, Australia; 9 QIMR Berghofer Medical Research Institute, Brisbane, Australia

**Keywords:** Neurotrauma, Neuroinflammation, Neuroimmunology, Immunosuppression, Neutrophil, Lymphocyte

## Abstract

Next-generation humanised mouse models and single-cell RNA sequencing (scRNAseq) approaches enable in-depth studies into human immune cell biology. Here we used NSG-SGM3 mice engrafted with human umbilical cord haematopoietic stem cells to investigate how human immune cells respond to and/or are changed by traumatic spinal cord injury (SCI). We hypothesised that the use of such mice could help advance our understanding of spinal cord injury-induced immune depression syndrome (SCI-IDS), and also how human leukocytes change as they migrate from the circulation into the lesion site. Our scRNAseq experiments, supplemented by flow cytometry, demonstrate the existence of up to 11 human immune cell (sub-) types and/or states across the blood and injured spinal cord (7 days post-SCI) of humanised NSG-SGM3 mice. Further comparisons of human immune cell transcriptomes between naïve, sham-operated and SCI mice identified a total of 579 differentially expressed genes, 190 of which were ‘SCI-specific’ (that is, genes regulated only in response to SCI but not sham surgery). Gene ontology analysis showed a prominent downregulation of immune cell function under SCI conditions, including for T cell receptor signalling and antigen presentation, confirming the presence of SCI-IDS and the transcriptional signature of human leukocytes in association with this phenomenon. We also highlight the activating influence of the local spinal cord lesion microenvironment by comparing the transcriptomes of circulating versus infiltrated human immune cells; those isolated from the lesion site were enriched for genes relating to both immune cell activity and function (e.g., oxidative phosphorylation, T cell proliferation and antigen presentation). We lastly applied an integrated bioinformatics approach to determine where immune responses in humanised NSG-SGM3 mice appear congruent to the native responses of human SCI patients, and where they diverge. Collectively, our study provides a valuable resource and methodological framework for the use of these mice in translational research.

**Supplementary Information:**

The online version contains supplementary material available at 10.1186/s12974-024-03048-0.

## Introduction

Spinal cord injury (SCI) triggers distinct systemic changes in circulating white blood cell (WBC) numbers and phenotype [1–3], and is associated with immune infiltration into the lesion site [4–6]. The inflammatory response itself has long been recognised as a therapeutic target in SCI, but translating and/or demonstrating the effectiveness of any such interventions clinically remains a challenge [6, 7]. Beyond heterogeneity in aetiology and clinical symptoms in the human patient population, cross-species differences may form an additional barrier to translation from animal models. Here we have characterised a next-generation NOD-SCID-gamma SGM3 (NSG-SGM3) humanised mouse model to determine whether it may serve as a useful tool in better understanding the human immune response following SCI.

Mice with ‘humanised immune systems’ are essentially immunocompromised hosts engrafted with human haematopoietic stem/progenitor cells (HSPCs), thereby allowing for the investigation of how human immune cells respond to various stimuli in the complex in vivo environment. Following engraftment, human HSPCs differentiate into many haematopoietic lineages, including human myeloid and lymphoid cells [8]. This potentially makes mice with humanised immune systems ideal models in which to study complex, finely choreographed immune responses. However, the advantageous features of these humanised mouse models have yet to be fully exploited for studying post-traumatic inflammation in SCI. Additionally, there is also a limited understanding as to what extent immune cell responses in these models accurately reflect those observed in human patients. In a previous generation humanised NSG (huNSG) mouse model, human immune cells were detected at the lesion site during the more intermediate-chronic phase of SCI (28–35 days post-injury; dpi), and their presence was associated with worsened outcomes [9, 10]. Here we chose to study NSG-SGM3 mice, a strain that was purposefully designed to improve human xenografting efficiency [11]. Similar to the NSG strain, NSG-SGM3 mice are non-obese diabetic (NOD), severe combined immunodeficient (SCID), and with a complete disruption in the interleukin-2 receptor (IL2R) common gamma-chain (IL2Rγ^null^), which eliminates host NK cells. NSG-SGM3 mice differ, however, from the NSG strain in that they additionally express select human transgenes for SCF-KIT ligand, GM-CSF/colony-stimulating factor 2 (CSF2) and IL-3 (SGM3), augmenting human myeloid lineage compositions [11, 12]. We theorised therefore that humanised NSG-SGM3 mice would be an ideal model for investigating the early inflammatory response to SCI, a phase that is yet to be fully explored in any humanised mouse strain and during which much immune cell infiltration occurs in both rodents and human patients [5, 13]. How human immune cells respond to and/or are altered by the local microenvironment once they reach the lesion site is also not yet understood.

In the present study, we have characterised how humanised NSG-SGM3 mice react to SCI, both in relation to early inflammation and also their longer-term recovery. We hypothesised that NSG-SGM3 mice would robustly support human myeloid cell development upon engraftment, and that the profiling of human leukocytes in these mice could provide clinically relevant insights into both spinal cord injury-induced systemic immune depression syndrome (SCI-IDS) and the influence of the local lesion microenvironment on their transcriptional state. Using histological approaches, flow cytometry and single-cell RNA sequencing (scRNAseq), we demonstrate the existence of 11 human immune cell types and/or states, both within the circulation and upon their entry into the lesion site, indeed identifying an immunosuppressive gene signature that is uniquely associated with and/or induced in response to SCI. We further compared these findings to a publicly available bulk RNAseq dataset from human patients to explore overlap. These data identify where immune responses appear congruent to native human responses and where they diverge, providing a methodological approach (with identified limitations) for the use of this mouse strain for translational research. Collectively, our studies offer novel insights into both convergence and divergence within the inflammatory response to SCI in mice with humanised immune systems.

## Methods

### Mice

**Fig. 1:**
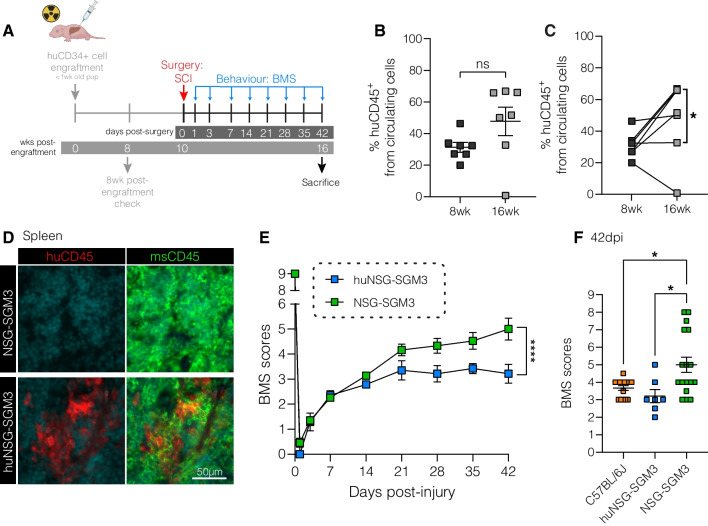
Humanised NSG-SGM3 mice have worse functional recovery from SCI compared to non-humanised controls. **A** Experimental timeline. NSG-SGM3 pups were irradiated and engrafted with human CD34 + (huCD34 +) stem cells. Mice received spinal cord injury (SCI) surgery at 10 weeks post-engraftment. Functional recovery was assessed weekly using the Basso Mouse Scale (BMS). **B** Percentage of human CD45 + (huCD45 +) out of all live circulating cells at 8 and 16 weeks-post engraftment (n = 7 per timepoint; 5♀, 2♂). **C** Comparison of the change in engraftment efficiency between individual mice. Asterisk highlights significant temporal increase in huCD45 + cell when removing one animal with engraftment failure (n = 7 per timepoint; 5♀, 2♂). **D** Representative immunofluorescent images of spleens from non-engrafted NSG-SGM3 and humanised NSG-SGM3 (huNSG-SGM3) mice, at 16 weeks post-engraftment, stained for huCD45 (red; A488), mouse CD45 (msCD45; green; A647) and Hoechst (cyan). Scale bar is 50 μm. **E** Pooled BMS scores for huNSG-SGM3 (blue; n = 7) and NSG-SGM3 (green; n = 23 (14♀, 9♂) for days 0–7 post-SCI; n = 17 (9♀, 8♂) for days 14–35 post-SCI) mice following SCI. **F** BMS scores at 42 days post-injury (dpi) for huNSG-SGM3 (n = 7; 5♀, 2♂), non-humanised NSG-SGM3 (n = 17; 9♀, 8♂), and C57BL/6 J mice (orange; n = 19; 15♀, 4♂). Data are mean ± SEM for all graphs, with statistical analysis being either a paired *t*-test (**B**, **C**), two-way ANOVA (**E**), or one-way ANOVA with Tukey’s post-hoc (**F**); *, *p* < 0.05; ****, *p* < 0.0001; ns, not significant

**Fig. 2:**
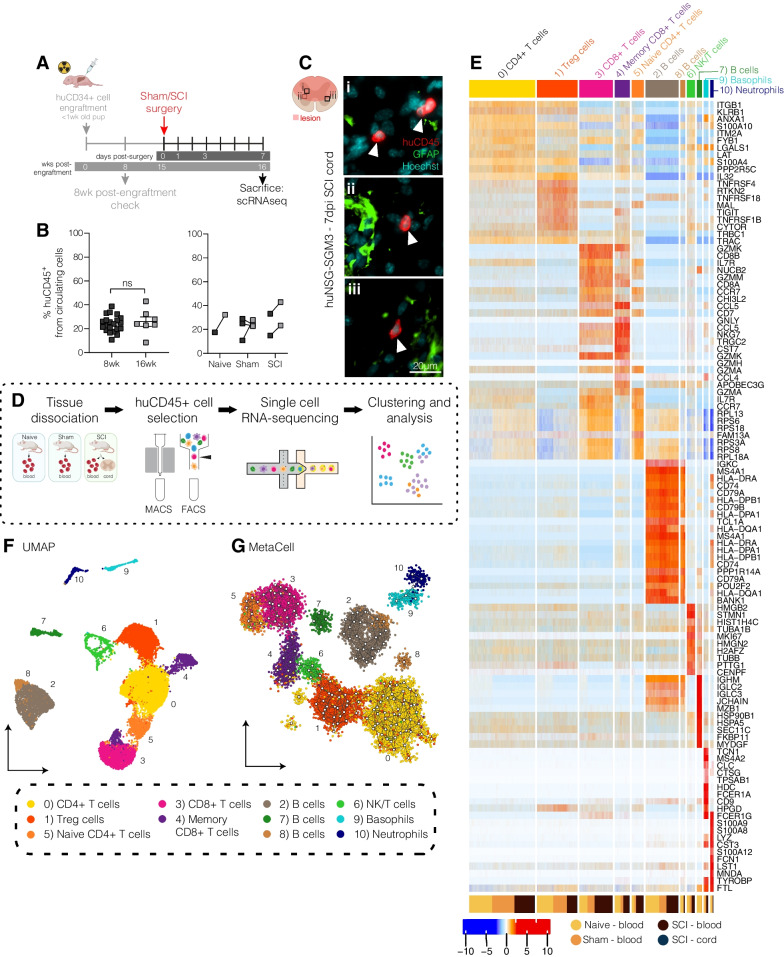
Profiling of the immune response to SCI in humanised mice. **A** Experimental timeline. huNSG-SGM3 mice were subjected to either laminectomy (sham), spinal cord injury (SCI), or no surgery (naïve controls) at 15 weeks post-engraftment and then sacrificed 7 days later. **B** Left: engraftment efficiency at 8- (n = 21; 7♀, 14♂) and 16-weeks post-engraftment (study endpoint; n = 7; 3♀, 4♂). Data points show individual animals, along with group means and standard error of the mean (SEM); unpaired *t*-test; ns, not significant. Right: Temporal change in engraftment efficiency for individual mice. Lines highlight the direction of change in engraftment efficiency over time. **C** Representative immunofluorescent images showing huCD45 + cells (red; A647) within the different areas of the lesion (i-iii in spinal cord cartoon) in huNSG-SGM3 mice with SCI (7dpi; scale bar is 20 μm); staining for glial fibrillary acidic protein (GFAP) is shown in green (A488) and cell nuclei are cyan. Arrows indicate huCD45 + cells. **D** Experimental workflow for single cell RNA sequencing (scRNAseq) experiments. huCD45+ cells were isolated by fluorescence activated cell sorting (FACS) from the blood and/or spinal cord for scRNAseq. **E** Heatmap showing the top-10 gene markers for each identified cell type; colour gradient shows the scaled expression for each cluster marker (centered data divided by the standard deviation). **F** UMAP of all immune cells collected and sequenced from blood and spinal cord samples. Cells were classified into 11 cell types/subsets based on marker expression. **G** MetaCell plot of all immune cells, coloured by cell type and subset

A total of 23 NSG-SGM3 (14♀, 9♂), 34 humanised NSG-SGM3 mice (13♀, 21♂) and 19 C57BL6/J (15♀, 4♂) age-matched mice were used in this study; all mice were obtained from the Animal Resource Centre (Canningvale, Western Australia) and/or local breeding colonies maintained by The University of Queensland’s Biological Resources facility. Mouse age at each procedural step (i.e., humanisation of the immune system and surgery) and endpoints are as specified below and/or in the experimental timelines (see Figs. 1A and 2A). Humanised (hu) NSG-SGM3 mice were generated as described previously [11, 14]. In brief, cord blood was obtained from the Queensland Cord Blood Bank. Density gradients were used to isolate CD34 + haematopoietic stem cells, followed by positive selection using a CD34 + isolation kit (Miltenyi Biotec). Next, two-to-five-day old NSG-SGM3 pups (NOD.Cg-Prkdc^scid^IL2rg^tm1Wjl^/Tg(CMV-IL3,CSF2,KITLG)1Eav/MloySzJ; stock no. 013062, The Jackson Laboratory) were conditioned using total body irradiation (100cgy), followed by intrahepatic injection of human CD34 + cells 4 h after irradiation (4.0 × 10^4^ cells for Cohort 1; n = 9 (5♀, 4♂) (Fig. 1A), and 4.2 × 10^4^ cells for Cohort 2; n = 21 (7♀, 14♂) (Fig. 2A)). All mice were allowed to acclimatise for at least 14 days prior to experimentation and housed in individually ventilated cages under specific pathogen-free conditions in certified animal holding facilities at the host institution. They were maintained on a 12-h light–dark cycle and had unlimited access to food and water. Two male mice, both from Cohort 1, were euthanised prior to study completion as they reached humane endpoints on general wellbeing score sheets (weight loss, ruffled fur and hunched posture); these mice were excluded from all subsequent analysis. We otherwise did not observe any indications for an increased rate of infectious complications and/or mortality in NSG-SGM3 and huNSG-SGM3 mice under our experimental paradigm.

### Surgical procedures

All surgeries were performed using aseptic technique in a UV-sterilised room. All solutions for injection (e.g., anaesthetic, saline) were either filter sterilised or drawn up in a sterile Biosafety Cabinet prior to injection. Mice for behavioural studies (Cohort 1) were 2–3 months of age at the time of surgery (Fig. 1A), and 3–4 months of age for Cohort 2 (Fig. 2A); mice were administered inhalable oxygen (50%) for a minimum of 5 min prior to anaesthetic injection.

Mice were anaesthetised with a combination of xylazine (20 mg/kg; intra-peritoneal (IP); Troy Laboratories) and tiletamine/zolazepam (50 mg/kg; IP; Virbac), followed by a thoracic contusive SCI as previously described [15]. In brief, paravertebral muscles over the target region were partitioned and anatomical landmarks used to identify T9 [16]. Following this, a dorsal laminectomy of T9 was performed and the spinal column clamped and stabilised. A severe (70kdyne) contusive SCI was then inflicted onto the exposed spinal cord segment using the Infinite Horizon Impactor (Precision Systems and Instrumentation) [17]. The actual applied force and associated tissue displacement were recorded post-impact for each animal, and not different between experimental groups and/or conditions (data not shown). Wounds were closed with 6–90 polygalactin dissolvable sutures (Ethicon) and Michel wound clips (Kent Scientific). Where relevant, sham-operated mice (i.e., ‘laminectomy only’) were also included to control for the effects of surgery itself. Naïve mice were not injured. All mice were kept in a Harvard Apparatus Small Animal Recovery Chamber to maintain body temperature during recovery from anaesthesia. For post-operative care, mice received a single dose of pain killer (buprenorphine in Hartmann’s sodium lactate; 1mkg/kg; sub-cutaneous (SC); Sigma Aldrich), immediately upon waking post-operation, and antibiotic treatment (gentamycin; 10 mg/kg/day; SC; Sigma Aldrich) once daily for 5dpi. Bladders of SCI mice were manually checked and voided twice daily for the duration of each experiment.

### Flow cytometry

Flow cytometry was used to characterise select immune cell populations as part of engraftment checks. For this, mice were anaesthetised with methoxyflurane (inhalation), and blood collected at 8 weeks post-engraftment via retro-orbital bleed into lithium-heparin tubes (BD Biosciences); study endpoint blood samples (16 weeks post-engraftment) were obtained via cardiac puncture. Blood samples were processed for flow cytometry as described below under ‘Blood processing’. Additional routine haematological analysis was performed (where specified) on the Mindray BC-5000 Vet Haematology Analyser, using blood samples collected at room temperature (RT) in ethylenediminetetraacetic acid (EDTA) tubes (BD Biosciences) and kept on ice until analysis.

For all other flow cytometry and/or cell sorting experiments, collection procedures were performed under general anaesthesia with sodium pentobarbitone (100 mg/kg; IP; Virbac) prior to processing. The thoracic cavity and pericardial sac were then opened to allow for transcardial perfusion. Where appropriate, blood was collected via cardiac puncture (detailed below). A syringe needle was then inserted into the left ventricle of the heart through the apex, after which the right atrium was punctured. Mice were perfused with 10–15 ml 1 × Hank’s Buffered Salt Solution without Ca^2+^ or Mg^2+^ (HBSS; Life Technologies) containing 0.2% heparin (Pfizer) prior to organ dissection.

### Blood processing

Blood was collected via retro-orbital bleed or cardiac puncture as described above. For collection via cardiac puncture, the heart was exposed under general anaesthesia by opening thoracic cavity, after which a heparinised syringe was inserted into the left ventricle chamber of the heart to draw up blood. Bloods were immediately transferred into lithium-heparin tubes (BD Bioscience). Next, 50 µl of blood was mixed with 150 µl of anti-coagulant buffer (4 mM EDTA; Sigma Aldrich) in Dulbecco’s phosphate-buffered saline (DPBS; 0.02% KCl, 0.02% KH_2_PO_4_, 0.8% NaCl, 0.1% Na_2_HPO_4_; 1:4 ratio), followed by incubation with 1 ml red blood cell (RBC) lysis buffer (1:5 ratio) for 7 min at RT. Samples were then centrifuged for 10 min at 300 × *g* (4 °C) and resuspended in 100 µl DPBS for staining.

### General antibody staining procedure for flow cytometry

Appropriate single cell suspensions in DPBS were incubated with viability dye (Zombie Green/NIR/Violet (1:100); BioLegend) for 20 min on ice. Alternatively, where required, samples were stained with propidium iodide (added immediately prior to analysis) rather than Zombie dye as the live/dead exclusion marker (1/3200; Thermo Fisher). Cells were then centrifuged at 4 °C for 10 min at 300 × *g* (blood) or 500 × *g* (spinal cord). Cell pellets were resuspended in FACS buffer, i.e., 0.5% Bovine Serum Albumin (BSA; Sigma Aldrich) and 2 mM EDTA in DPBS, followed by a 10-min incubation with anti-CD16/32 (1:200; BD Bioscience) for Fc receptor blocking. Next, samples were incubated with an appropriate cocktail of fluorophore-conjugated antibodies (see Table 1) for 20 min (4 °C) and topped up with 1 ml FACS buffer. Samples were then centrifuged again for 10 min at 4 °C (300–500 × *g*) and cell pellets gently resuspended in FACS buffer. Propidium iodide-fluorescing counting beads (5 µl; Beckman Coulter) were then added to each tube as an internal standard to allow for accurate calculation of absolute cell numbers. Samples were analysed on the Fortessa flow cytometer (BD Biosciences) with BD FACS Diva software, and data was analysed with FlowJo software (v10.8, Tree Star, Inc.). Single-stain compensation beads (ABC Total Antibody Compensation Bead Kit; Thermofisher) were included to allow for removal of spectral overlap between fluorophores during the analysis phase.

**Table 1:** Antibodies used for immunofluorescence and flow cytometry

Antibody	Concentration
*Immunofluorescence primaries*	
Rabbit anti-human CD45 (clone EP322Y, #AB40763, Abcam)	1/200
Goat anti-mouse CD45 (polyclonal, #AF114, R&D Systems)	1/400
Chicken anti-mouse GFAP (polyclonal, #AB4674, Abcam)	1/400
*Immunofluorescence secondaries and nuclear dye*	
Alexa Fluor® 488 Donkey Anti-Human IgG (Fcγ-specfic) (polyclonal, #709545098, Jackson ImmunoResearch)	1/100
Alexa Fluor® 488 AffiniPure F(ab')_2_ Fragment Donkey Anti-Chicken IgY (IgG) (H + L) (polyclonal, #703546155, Jackson ImmunoResearch)	1/400
Alexa Fluor® 488 AffiniPure F(ab')_2_ Fragment Donkey Anti-Rabbit IgG (H + L) (polyclonal, #711546152, Jackson ImmunoResearch)	1/400
Alexa Fluor® 647 AffiniPure F(ab')_2_ Fragment Donkey Anti-Rabbit IgG (H + L) (polyclonal, #JI711606152, ImmunoResearch)	1/400
Alexa Fluor® 647 AffiniPure F(ab')_2_ Fragment Donkey Anti-Goat IgG (H + L) (polyclonal, #JI705606147, Jackson ImmunoResearch)	1/400
Hoechst 33342 Trihydrochloride Trihydrate (H3570, Invitrogen)	1/1000
*Flow cytometry*	
Anti-human CD14 BV711 (clone MPɸ9, #563372, BD)	1/200
Anti-human CD45 APC-Cy7 (clone 2D1, #368516, Biolegend)	1/50
Anti-mouse CD45 PE (clone 30-F11, #553081, BD)	1/100
Anti-mouse/human B220 AF700 (clone RA3-6B2, #103232, Biolegend)	1/25
Anti-mouse/human CD11b BV786 (clone M1/70, #101,243, Biolegend)	1/400
Propidium iodide (#P1304MP, Life Technologies)	1/3200
Anti-Cy7 MicroBeads (#130–091-652, Miltenyi Biotec Australia)	

### Behavioural analysis

The Basso Mouse Scale (BMS) was used to evaluate locomotor performance of SCI mice at regular intervals post-surgery. Mice were observed in an open field (a circular, flat area) for 4 min by at least 2 single-blinded investigators. Published guidelines [18] were used to rate individual mice on a scale of 0–9 (9 indicating normal locomotion) based on their hindlimb movement, stepping ability, fore-hindlimb coordination and trunk/tail stability.

### Tissue processing for histology

For histological assessment, mice were perfused with 10-15 ml saline containing 2% NaNO_2_ and 10 IU/ml heparin (Pfizer), to prevent coagulation, followed by 20-30 ml of phosphate-buffered Zamboni’s fixative (2% picric acid, 2% paraformaldehyde, pH 7.2–7.4). Next, spleens, livers and vertebral columns were removed and post-fixed overnight at 4 °C. Spinal cords were then dissected from the vertebral column on the following day and again post-fixed overnight. They were then cryoprotected along with other organ samples by immersion in 10% and then 30% sucrose solution at 4 °C. Following this, all tissue samples were embedded in Optimal Cooling Temperature compound (ProSciTech) and snap-frozen in methylbutane on dry ice. Transverse spinal cord, liver and spleen sections (20 µm) were cut using a Leica Cryostat CM3050-5, collected onto Superfrost Plus slides (1:5 series; Lomb Scientific), air-dried and then stored at −80 °C until further processing.

### Immunofluorescent staining

For visualisation of select immune cell populations within the injured spinal cord (see Table 1), slides were defrosted and air-dried for 60 min at RT, after which they were washed in 1 × phosphate-buffered saline (PBS). Slides were then incubated for 1 h at RT in blocking buffer (2% BSA, 0.2% Triton X-100 in PBS) to reduce non-specific binding. Following this, they were washed again in PBS and incubated overnight at 4 °C with an appropriate cocktail of primary antibodies (diluted in blocking buffer). The following day, slides were washed again in PBS and incubated for 1 h at RT with secondary antibodies as well as Hoechst 33342 nuclear dye (1:1000; Sigma Aldrich). After a final round of washing, slides were cover slipped with DAKO fluorescent mounting medium (Sigma-Aldrich).

Stained spleen (Fig. 1D) and spinal cord sections (Fig. 2C) were imaged using a high speed automated Nikon sterology upright widefield fluorescence microscope with a 40 × air objective (Plan APO, numerical aperture 0.95, working distance 0.17–0.25 mm) and Hamamatsu Orca Flash 4.0 sCMOS camera (2048 × 2048 pixels, 6.5 × 6.5 μm pixel size, 82% QE). For Additional file 1: Fig. S4A-C, images were taken on a Diskovery Spinning Disk Inverted Confocal Microscope using a 70 μm spinning disk, 20 × air objective (CFI Plan APO VC, numerical aperture 0.75, working distance 1.00 mm) and Zyla 4.2 sCMOS camera (2048 × 2048 pixel resolution, 6.5 × 6.5 μm pixel size, 82% QE). All images were acquired at RT. NIS Elements Advanced Research software was used for image acquisition and brightness and contrast display settings were adjusted post-acquisition using ImageJ (v1.52p; display settings were made the same between images within a single figure panel).

### H&E staining and GVHD assessment

For haematoxylin and eosin (H&E) stains, liver sections were stained with Mayer’s haematoxylin for 4 min, washed in running water for 2 min, followed by 70% EtOH for 2 min, and then stained with 80% ethanol-eosin for 7 s. Next, samples were again washed in 90% EtOH, followed by 100% EtOH (repeated three times), before being cleared in xylene, mounted with DepeX and then coverslipped. Sections were imaged using a Zeiss AxioScan Z1 slide scanner at 20 × magnification.

Graft-versus-host disease (GVHD) pathology in H&E stained liver sections was manually quantified by an investigator who was blinded to the experimental condition, using established grading criteria for the degree of mononuclear infiltrate [19, 20]. A score of 0 reflected no visible pathology, escalating from here with a score of 1 being defined as punctate infiltration of mononuclear cells, 2 as sporadic perivascular infiltration of mononuclear cells, 3 as sporadic perivascular infiltration of mononuclear cells with some additional infiltration of the parenchyma, or 4 as widespread perivascular infiltration of mononuclear cells with spread into the parenchyma.

### Spinal cord processing for cell sorting

After perfusion with HBSS, the T8-T10 region of the spinal cord was dissected and weighed in an Eppendorf tube containing 500 μl HBSS (Life Technologies). Spinal cords were then mechanically dissociated using fine scissors, pelleted (10 min at 500 × *g*; 4 °C), and enzymatically digested for 20 min at 37 °C in 2.5 ml L-glutamine-containing Leibovitz’s L-15 Medium (Life Technologies) containing 0.1% papain (Worthington) and 0.1% DNase I (Roche). Next, spinal cord samples were triturated by gently pipetting the solution up and down (10–15 times), before being passed through a 40 μm cell strainer with 5 ml neutralising solution (10% Fetal Bovine Serum (FBS; Sigma Aldrich) in Dulbecco’s Modified Eagle Medium (Life Technologies)). Cell suspensions were then centrifuged for 10 min at 500 × g and resuspended in 100 µl DPBS for live/dead and antibody staining as detailed under ‘General antibody staining procedure for flow cytometry’.

Following this, spinal cord samples were additionally incubated with 20 µl anti-Cy7 MACs beads (Miltenyi Biotec) in 80 µl FACS buffer for 15 min at 4 °C, washed in 1 ml FACS buffer at 500 × *g*, and then run through MS Columns (Miltenyi Biotec) as per the manufacturer’s instructions to allow for positive selection/enrichment of human CD45 + (huCD45 +) cells prior to cell sorting.

### Sample preparation and sorting for single-cell RNA sequencing

Male naïve and sham-operated huNSG-SGM3 controls (n = 1 technical replicate per condition) as well as huNSG-SGM3 SCI mice (n = 2 technical replicates), all from Cohort 2, were deeply anaesthetised with 4% isoflurane at 7dpi. Blood was collected via cardiac puncture from all mice, in addition to the T8-T10 spinal cord segments from SCI mice. All samples were obtained and swiftly processed on ice and/or at 4 °C as detailed earlier; a minimum of 350 µl blood was taken from each mouse and added volumes of 4 mM EDTA and RBC lysis buffer adjusted on an individual basis to account for any variations in sampled blood volumes.

Next, Fc receptors were blocked with mouse IgG (1 μg/ml in FACS buffer, Jackson) for 10 min, followed by incubation of the samples with anti-human CD45 APC-Cy7 (1:50, clone 2D1; BioLegend) on ice for 20 min. Spinal cord samples were additionally incubated with 20 µl anti-Cy7 MACs beads (Miltenyi Biotec) in 80 µl FACS buffer for 15 min at 4 °C. Spinal cord samples were then washed, passed through an MS MACS column (Miltenyi Biotec) and huCD45 + cells collected as per the manufacturer’s instructions. All samples were resuspended in 400 µl FACs buffer and stained, as appropriate, with propidium iodide for live/dead exclusion (1:400; Thermofisher) prior to fluorescence activated cell sorting (FACS); single-stained samples were included to allow for the removal of any spectral overlap between fluorophores. huCD45 + cells were sorted using a BD FACS Aria Cell Sorter, with all samples gated to exclude debris, doublets and triplets, and dead cells. huCD45 + events were sorted into sterile RNAse-free Eppendorf tubes containing 500 $\upmu$l sterile, ice-cold PBS + 10% FBS (Additional file 1: Fig. S3; orange gate).

### *RNA sequencing library preparation and Chromium 10* × *single cell 3’ v3 sequencing*

Library preparation and sequencing was performed at the Institute for Molecular Bioscience Sequencing Facility (The University of Queensland). Here, a cell count was performed first to determine cell viability (93–100%) and yield post-sorting. Single-cell suspensions were partitioned and barcoded using the 10X Genomics Chromium Controller (10X Genomics) and the Single Cell 3' Library and Gel Bead Kit (V3; 10X Genomics; PN-1000075 or PN-1000092). The cells were loaded onto the Chromium Single Cell Chip B (10X Genomics; PN-1000073 or PN-1000074) to target a total of 20,000 cells per sample. GEM generation and barcoding, cDNA amplification, and library construction were all performed according to the 10X Genomics Chromium User Guide. A total of 11 cDNA amplification cycles were performed, and one quarter of the cDNA was used as input for library construction. 12 SI-PCR cycles were used for final indexing PCR. Reactions were performed in a C1000 Touch thermal cycler with a Deep Well Reaction Module (Bio-Rad).

Single cell transcriptome libraries were quantified on the Agilent BioAnalyzer 2100 using the High Sensitivity DNA Kit (Agilent, 5067-4626). Gene expression libraries were pooled in equimolar ratios. The final pool was quantified by qPCR using the KAPA Library Quantification Kit—Illumina/Universal (KAPA Biosystems, KK4824) in combination with the Life Technologies Viia 7 real time PCR instrument. Denatured libraries were loaded onto an Illumina NextSeq-500 and sequenced using a 150-cycle High-Output Kit as follows: 28 bp (Read1), 8 bp (i7 index), 111 bp (Read2). Read1 supplies the cell barcode and UMI, i7 the sample index, and Read2 the 3’ sequence of the transcript. All samples were processed in one sequencing run.

### scRNAseq analysis

Raw sequencing reads were processed and then mapped to a custom reference genome combining the human (GRCh38) and mouse (mm10) genomes in CellRanger v3.1.0 (10X Genomics). Cells were computationally divided into human and mouse fractions using k-means clustering (k = 2), with inspection of the gene content for the two clusters confirming the successful splitting of cells by species (data not shown). Cells matching the mouse genome were discarded and the resulting filtered count matrices from human cells used for downstream analysis. Median absolute deviation (MAD) filtering was implemented using scater v1.14.6 [21] to remove cell outliers with low library size and/or gene counts (> 3 MADs below the median value), or high mitochondrial or ribosomal gene percentage (> 3 MADs above the median value). Doublets were predicted with scds v1.2.0 [22] and cells were removed if they were predicted as doublets by at least two of the three included prediction methods (bcds, cxds, or hybrid), and expressed more than 3,000 genes. Gene counts were normalised using scran v1.14.6 [23]. All blood and spinal cord datasets were integrated for downstream analysis. Data scaling, dimensionality reduction, clustering and sub-clustering, data integration, and marker prediction were all performed in Seurat v3.1.4.9904 or Seurat v4.0.0 [24, 25]. Seurat objects were converted to SingleCellExperiment v1.8.0 objects where required [26]. Different cluster resolutions were tested and assessed using Clustree v0.4.2 [27]; a final resolution of 0.4 was selected after considering cluster stability, clustering patterns and top marker genes. Clusters were named based on assessment of top marker genes. The MetaCell pipeline [28] was implemented using default parameters. Differentially expressed genes (DEGs) were detected using Seurat, and visualised using ComplexHeatmap v 2.6.2 [29]. Gene ontology (GO) analyses were performed using the hypergeometric test in ClusterProfiler [30, 31] against the org.Hs.eg.db database v3.12.0 [32], and using a gene background as the union set of only genes expressed within each dataset. Ribosomal and mitochondrial genes were removed before DEG and GO analysis. Cell–to–cell interaction (CCI) analyses were run using CellChat v1 [33], with the built-in human interaction database; subsequent GO analyses were performed above using a gene background of expressed ligands or receptors only, as found in the CellChat database. All analyses were run on R v4.0.3 using a Benjamini-Hochberg-adjusted *p*-value of less than 0.05 as the significance threshold.

Finally, for comparisons of our in-house scRNAseq dataset from humanised mice with a publicly available bulk RNASeq dataset from human SCI subjects [1], we first log-transformed the counts for both datasets and averaged each gene. A linear model was then fitted over these pairs of average expression values for each gene, and a linear regression performed to identify correlated expression patterns between datasets. This was also repeated for each comparable condition between our data and the Kyritsis data, i,e, naïve mice against healthy human control subjects (HC), sham-operated mice against the human trauma control (TC) patients, and between spinal cord-injured (SCI) mice and human patients.

### Statistical analysis

Statistical analysis for behavioural assessment, histology and flow cytometry experiments was conducted using either a student’s T-test, one- or two-way ANOVA with post-hoc corrections for multiple comparisons as appropriate. Group sizes for engraftment checks and behavioural studies were based on relevant previous experiments and the literature [9]. Our scRNAseq dataset was otherwise sufficiently large to detect rare cell (sub)types (as few as 5 cells) with over 99% sensitivity and a false discovery rate of less than 5% [34]. All data are presented as mean ± standard error of the mean (SEM), with statistical significant results defined as p < 0.05. For all graphs, *p < 0.05, **p < 0.01, ***p < 0.005 and ****p < 0.0001.

## Results

### Improved SCI recovery of NSG-SGM3 mice is lost with humanisation

We first confirmed the long-term engraftment of human immune cells in our huNSG-SGM3 mouse model. In brief, immunodeficient (Additional file 1: Fig. S1) NSG-SGM3 pups were irradiated and administered CD34 + haematopoietic stem cells from human cord blood via intrahepatic injection, 2–4 days after birth. Mice then received SCI surgery at 10 weeks post-engraftment and were sacrificed 6 weeks later (i.e., 42 days post-SCI; Fig. 1A); blood samples were taken at 8 and 16 weeks post-engraftment, at which time there is typically stable human immune system development [9]. The percentage of circulating human WBCs (huWBCs) increased from 31.3 ± 3% at 8 weeks to 47.7 ± 9% (median ± SEM; p = 0.09) at 16 weeks post-engraftment (Fig. 1B). A comparison of the temporal fold-change in engraftment efficiency for individual mice confirmed that most animals had an increased percentage of huCD45 + cells at 16wks compared to 8wks (Fig. 1C), and the removal of one animal with engraftment failure (0.76% at 16 weeks post-engraftment) indeed demonstrated a significant expansion of human immune cells over time (p = 0.021; paired t-test). Flow cytometry confirmed that most circulating human cells were CD11b- lymphocytes, with the majority being B220- cells (likely T cells; 66.2 ± 11% of all huCD45 + cells) and B220 + B cells (27.5 ± 8% of all huCD45 + cells). A small population of circulating human CD11b + myeloid cells, including huCD14 + monocytes/neutrophils (0.7 ± 0.2% of huCD45 + cells) and huCD14- cells, was also detected (3.5 ± 1.7% of huCD45 + cells; Additional file 1: Fig. S2A, B). In contrast, endogenous mouse immune cells were mostly msCD11b + myeloid cells (99.1 ± 0.4%; Additional file 1: Fig. S2A, C). Human immune cells could also be detected in the spleens of huNSG-SGM3 (Fig. 1D), where they are known to properly organise themselves [12], and diffusely throughout the injured spinal cord (see below and Fig. 2C).

We lastly used open-field locomotor scoring (BMS) [18] to understand how the introduction of a human immune system influenced SCI recovery of NSG-SGM3 mice. huNSG-SGM3 mice recovered significantly less stepping ability overall compared to their non-humanised, immunodeficient (NSG-SGM3) counterparts (Fig. 1E). This finding is consistent with observations from other studies that fully immunocompetent strains typically show reduced recovery [9, 38], pointing to a net negative role of aberrant inflammation post-SCI in relation to outcomes. Endpoint analysis indeed confirmed that BMS scores of NSG-SGM3 were significantly higher at 42dpi than those of both huNSG-SGM3 and wild-type C57BL6/J strains (Fig. 1F).

### scRNAseq identifies human immune cell diversity in huNSG-SGM3 mice

Having confirmed long-term engraftment in huNSG-SGM3, we next turned to scRNAseq to investigate how human immune cells respond to and/or are affected by SCI. For this, a separate cohort of huNSG-SGM3 mice were engrafted as detailed earlier, subjected to sham (i.e., laminectomy only; trauma control) or SCI surgery at 15 weeks post-engraftment, and then sacrificed one week later (Fig. 2A). A comparison of engraftment efficiency in a random sample of animals from each experimental group, including pre- and post-surgery, once again showed that most mice displayed an increase in human immune cell content between 8 and 16wks post-engraftment; we also confirmed that there was no apparent effect of sham or SCI surgery on engraftment efficiency compared to naïve controls (Fig. 2B**)**.

We then used FACS sorting to isolate human immune cells from the blood of naïve and sham-operated controls as well as SCI mice, in addition to huCD45 + cells from the injured spinal cord itself (Fig. 2C, D and Additional file 1: Fig. S3); immune cells were not collected from the spinal cords of naïve and sham-operated mice as there was no inflammatory infiltrate in these animals (data not shown). Using the Chromium 10X platform, a total of 23,273 single-cell transcriptomes were obtained following data integration and quality control. Unsupervised clustering using the Seurat Louvain method [24] classified these into 11 clusters; cell cluster labels were identified based on the top differentiating genes and previously established canonical immune cell markers (Fig. 2E and F). They included: CD4 + T cells, represented across three clusters (CD4 + T cells (cluster 0; cl0), Treg cells (cl1) and naïve CD4 + T cells (cl5)). CD8 + T cells were represented across two clusters, namely CD8 + T cells (cl3) and memory CD8 + T cells (cl4). Three clusters of B cells were identified (clusters 2, 7 and 8) as well as two myeloid cell clusters (neutrophils (cl10) and basophils (cl9)), and a small population of NK/T cells (cluster 6). Overall, these cell type inferences match and/or were validated by independent flow cytometry data (Additional file 1: Fig. S2B). Single-cell transcriptomes were also partitioned using MetaCell, a graph network-based approach where cells are grouped into metacells and linked based on their transcriptional similarity [28]. The MetaCell analysis confirmed Seurat clustering results, with major related cell types appearing as colocalised and/or linked within the MetaCell network diagram (Fig. 2G). Human lymphoid lineages otherwise appeared functional, with splenic B cells expressing various immunoglobulin genes and demonstrating the ability to class-switch/produce human IgG (Additional file 1: Fig. S4A-D). CD8 + T cell and NK/T cell clusters were similarly found to express various effector (granzyme and perforin) genes **(**Additional file 1: Fig. S4E).

### Non-neurological tissue damage induces inflammation within the circulation

**Fig. 3:**
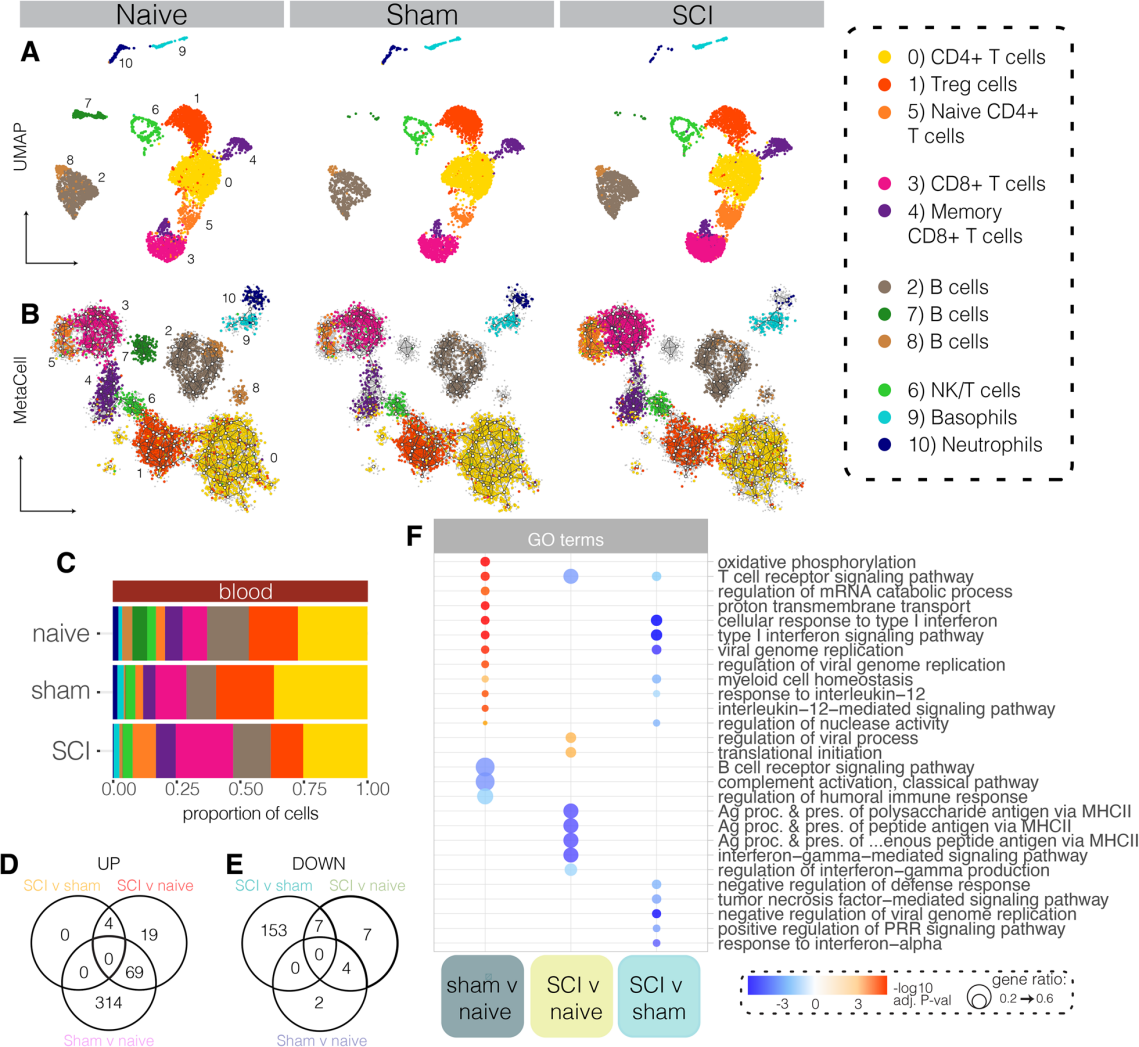
Comparison of immune cell type and states in the blood of humanised naïve, sham and SCI mice. **A** UMAP plots showing all cells present within naïve (left), sham (middle) and SCI (right) scRNAseq blood samples at 7 days post-surgery. **B** Metacell networks showing the clustering and/or colocalisation of all cells present within the naïve, sham and SCI scRNAseq blood samples (see also Additional file 1: Fig. S5). **C** Proportion of each cluster out of all cells present for each condition. **D** and **E** Number of differentially expressed genes (DEGs) up- (**D**) or down- (**E**) regulated between conditions. Overlap indicates genes that were regulated in the same direction between conditions. **F** Gene ontology (GO) terms that were differentially regulated between sham v naïve, SCI v naïve, or SCI v sham conditions. For GO plot, red colour represents –log10(adjusted *p*-value) for upregulated genes/terms, and blue colour the log10(adjusted *p*-value) for downregulated genes/terms; dot size represents ratio of genes contributing to the GO term

Having established the human immune cell repertoire of our huNSG-SGM3 mice, we next aimed to understand how these cells change within the circulation in response to trauma; that is, tissue injury itself but not necessarily SCI-specific responses. For this, we compared blood samples from sham (laminectomy only) to those of naïve and SCI mice. A total of 4,530 single-cell transcriptomes were analysed for sham, 6,224 for naïve, and 5,865 for the SCI condition. Projection of these onto either UMAP space or MetaCell networks revealed no major changes in cluster presence between conditions (Fig. 3A, B and Additional file 1: Fig. S5). A comparison of cell type abundance revealed that B cell cluster 7 was much reduced under sham (0.3%) and SCI (0.2%) conditions compared to naïve huNSG-SGM3 controls (5.8%); CD8 + T cells (cl3) were expanded in SCI (22.5%) compared to both naïve (9.7%) and sham (12.1%), as were naïve CD4 + T cells (cl5; 9.2% in SCI compared to 3.5% in naïve and 3.0% in sham) (Fig. 3C).

Comparison of gene expression between all three conditions (after removing ribosomal and mitochondrial genes) revealed a total of 579 DEGs between either SCI, sham or naïve conditions. These were then further split into those changed between sham and naïve (383 upregulated, 6 downregulated), SCI versus naïve (92 up, 18 down), or SCI versus sham (4 up, 160 down; Fig. 3D, E). Testing the association of these DEGs with functional GO terms revealed that the sham-operated group mostly exhibited an upregulation of inflammatory functions compared to the naïve group (Fig. 3F). This included increases in genes driving T cell receptor signalling, cellular responses to Type I interferon, responses to IL-12 and IL-12 signalling. These changes were spread across all cell types within the sham sample (Additional file 1: Fig. S6A, B). A separate collection of GO terms was decreased under both sham and SCI conditions compared to naïve controls (Additional file 1: Fig. S6C); these were termed ‘trauma-associated’ GO terms, as they were regulated by trauma itself rather than neurological injury.

In sharp contrast to our findings in sham-operated mice (i.e., non-neurological trauma controls), human immune cells in huNSG-SGM3 SCI mice mostly downregulated inflammatory functions compared to either naïve or sham samples (Fig. 3F). Downregulated GO terms in huNSG-SGM3 SCI mice versus naïve controls related to T cell receptor signalling, antigen processing and presentation via MHC Class II and interferon-gamma-mediated signalling pathways. Additional comparisons between SCI and sham groups similarly revealed (further) decreases in the expression of genes relating to various immune functions, including the downregulation of T cell receptor signalling, Type I interferon responses, responses to IL-12, TNF signalling, IFNα signalling and pattern recognition receptor signalling pathways. These findings indicate that while non-neurological tissue injury in sham-operated mice triggered a systemic inflammatory response, there was a failure and/or strongly diminished capacity within circulating human immune cells to do so after SCI, as evident from the downregulation of numerous immune-related genes and associated GO terms compared to both sham and naïve huNSG-SGM3 mice. Accordingly, communication between circulating immune cells via known ligand-receptor (LR) pairs was mostly reduced under SCI conditions, both in terms of number and strength of the predicted interactions (Additional file 1: Fig. S6D, E). The only observed increase in communication under SCI conditions relative to both naïve and sham-operated controls was between neutrophils and cluster 2 and 8 B cells (Additional file 1: Fig. S6E), with the associated GO terms linked to immune activation and chemotaxis (Additional file 1: Fig. S6F). Immune cell interactions in sham-operated mice were otherwise generally increased relative to naïve controls, as expected, with the only exception being the signalling strength from memory CD8 + T (cl4) and NK/T (cl6) cells to cluster 7 B cells (the presence of which was reduced under both sham and SCI conditions) (Additional file 1: Fig. S6E).

### The systemic SCI-specific response in humanised mice shows immunosuppression

**Fig. 4:**
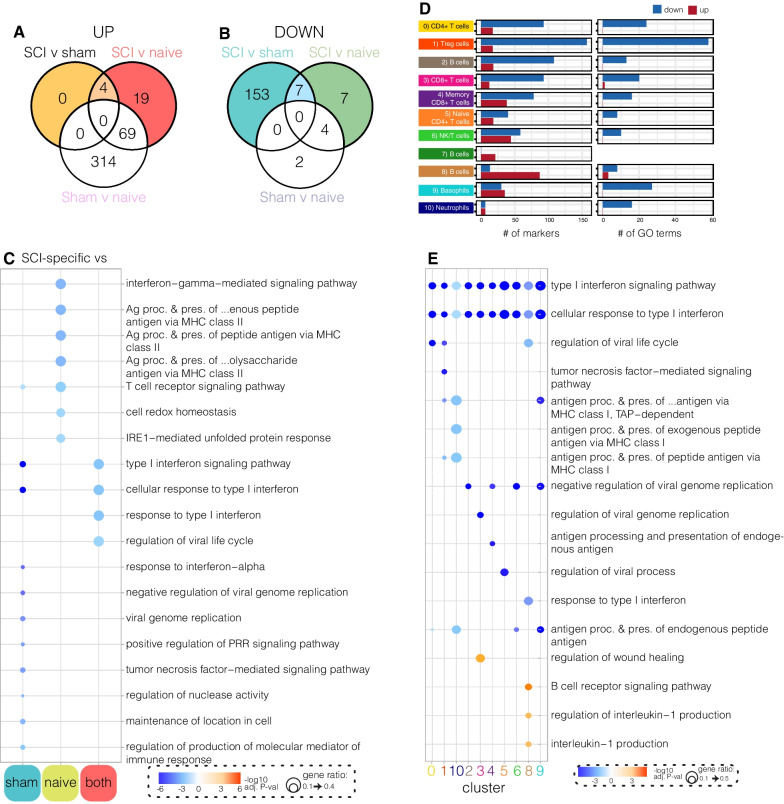
The SCI-specific gene response links to immunosuppression within the blood. **A** and **B** Number of differentially expressed genes (DEGs) up- (**A**) or down- (**B**) regulated between blood samples. Coloured parts of Venn diagram indicate SCI-specific genes (i.e., those regulated in the same direction by SCI compared to sham and/or naïve samples, but not between sham v naïve). **C** Unique statistically enriched gene ontology (GO) terms for the SCI-specific gene set. GO terms are split based on whether they were regulated in SCI compared to either sham, naïve, or both. **D** Number of gene markers (i.e., DEGs; left) and GO terms (right) regulated in each cell cluster following SCI compared to both sham and naïve controls. **E** Top GO terms between clusters for genes regulated in SCI compared to both sham and naïve conditions. Red colour in GO plots represents –log10(adjusted *p*-value) for upregulated genes/terms, and blue colour the log10(adjusted *p*-value) for downregulated genes/terms; dot size represents ratio of genes contributing to the GO term

To further characterise the response to neurological injury, we next identified the genes that were changed only after SCI and not trauma controls (i.e., sham mice). Of the 579 DEGs within our dataset, 190 were identified as SCI-specific genes. Of these, 23 were upregulated and 167 downregulated by SCI compared to sham and/or naïve conditions (coloured parts of Venn Diagrams; Fig. 4A, B). These SCI-specific DEGs were then further split into whether they were altered *a)* in response to sham surgery, *b)* relative to naïve controls, or *c)* compared to both sham and naïve (control) conditions. The top-10 GO terms included in the SCI-specific response relative to sham surgery included a downregulation of GO terms relating to Type I interferon signalling, response to interferon-alpha and TNF signalling, and regulation of pattern recognition receptor signalling (Fig. 4C). Those included in the SCI-specific response compared to naïve (homeostatic) conditions included a downregulation of genes associated with IFN-gamma signalling, antigen processing and presentation via MHC Class II, and T cell receptor signalling, as well as cellular redox homeostasis and dealing with endoplasmic reticulum stress. Finally, four shared downregulated GO terms were found under SCI conditions compared to both sham and naïve controls; all of these related to Type I interferon signalling.

We next investigated how the identified SCI-specific changes in gene expression related to individual cell clusters (Fig. 4D). DEGs, mostly downregulated ones, and associated GO terms were identified in virtually all cell types, with the exception of cluster 7 B cells which were mostly absent in response to injury (i.e., under both sham and SCI conditions; see Fig. 3A, B); the fate of these cells remains unknown. Decreases in Type I interferon signalling, and cellular responses to Type I interferon, were identified within every other cell type/cluster (Fig. 4E). GO terms associated with antigen processing and presentation were downregulated within clusters 2 (B cells), 8 (B cells), 9 (basophils) and 10 (neutrophils). The only GO terms that were upregulated systemically in response to SCI were regulation of wound healing in the memory CD8 + T cell cluster (cl3; key driver genes *ACTG1*, *CXCR4*, *FCER1G* and *ANXA1* have all been previously associated with T cell activation and/or recruitment), B cell receptor signalling and IL-1 production within the B cell cluster (cl8).

Together, this data shows a prominent and broad decrease in the functional status of human immune cells after SCI based on transcriptome analysis, and identified a set of 190 genes in huNSG-SGM3 mice that were specifically regulated in this condition.

### The transcriptional state of infiltrated human immune cells is shaped by the lesion microenvironment

**Fig. 5:**
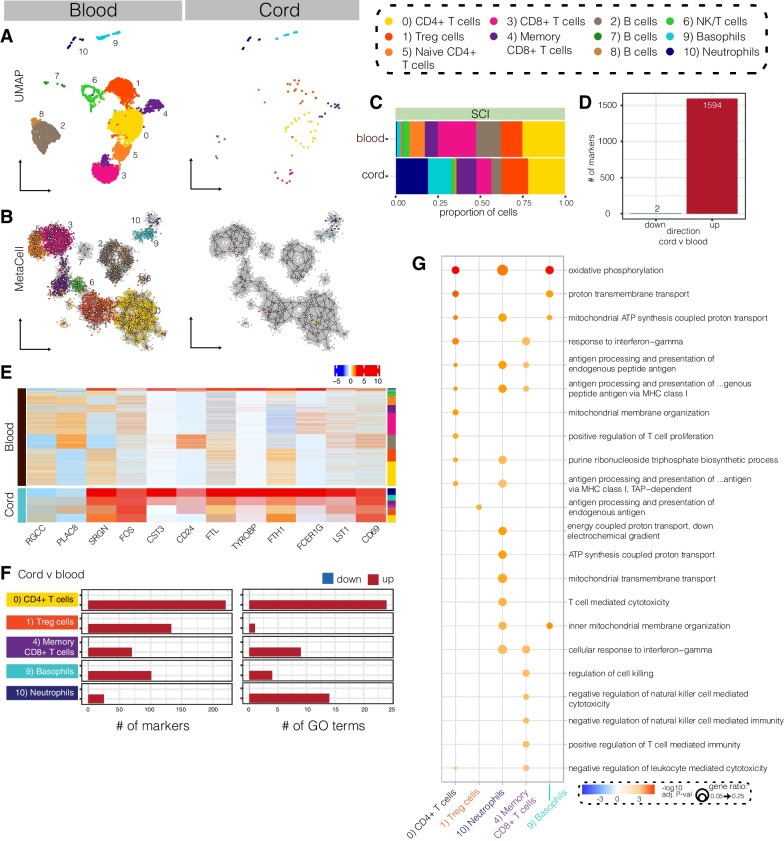
Comparison of single-cell transcriptomes from circulating and infiltrated human immune cells highlights the influence of the lesion microenvironment on cell states. **A** and **B** Annotated UMAP (**A**) and MetaCell (**B**) plots of all cells present within SCI blood (left) and cord (right) samples. **C** Proportion of cell type / clusters out of all cells present. **D** Number of differentially expressed genes (DEGs) either down- or upregulated (red) in human immune isolated from the spinal cord versus blood. **E** Heatmap showing gene expression of the top-10 genes that were either down- or upregulated in human immune cells that were isolated from the injured spinal cord. **F** Number of DEGs (i.e., markers; left) and GO terms (right) for each cell cluster between compartments (i.e., cord v blood). Blue represents terms downregulated and red represents terms upregulated in the huCD45 + cells from spinal cord compared to blood samples. **G** Top GO terms enriched for each cluster. Red colour in GO plot represents –log10(adjusted *p*-value) for upregulated genes/terms, while blue colour the log10(adjusted *p*-value) would represent downregulated genes/terms; dot size represents ratio of genes contributing to the GO term

Having characterised the systemic inflammatory response to SCI in huNSG-SGM3 mice, we next sought to understand how human immune cells change once they enter into the injury site, something that cannot be achieved in human patients. For this, a total of 101 high quality single-cell transcriptomes isolated from the injured spinal cord were compared with their circulating counterparts. Integration of these into our blood scRNAseq atlas identified these cells, in order of prominence, as CD4 + T cells (cluster 0; 21.8%), neutrophils (cluster 10; 18.8%), Treg cells (cluster 1; 15.8%), basophils (cluster 9; 13.9%), memory CD8 + T cells (cluster 4; 11.9%), CD8 + T cells (cluster 3; 8.9%), B cells (cluster 2; 5.9%), NK/T cells (cluster 6; 2.0%), and naïve CD4 + T cells (cluster 5; 1.0%) (Fig. 5A–C). In total, only 2 DEGs were downregulated (compared to blood data) in human immune cells that were isolated from the injured spinal cord, namely *RGCC* and *PLAC8* (Fig. 5D, E), while 1594 DEGs (Additional file 1: Fig. S**7A**) were upregulated and linked to an increase in inflammatory GO terms (Fig. 5D and Additional file 1: Fig. S7B); expression of the top-10 DEGs is shown in Fig. 5E and Additional file 1: Fig. S7A.

Limited by the number of high-quality human immune cell transcriptomes obtained from the injured spinal cord, downstream analysis of individual clusters was focused only on clusters containing > 10 cells; which included clusters 0 (CD4 + T cells), 1 (Treg cells), 4 (memory CD8 + T cells), 9 (basophils) and 10 (neutrophils). DEGs and associated enriched GO terms between infiltrated versus circulating immune cells were identified for each of these populations; all of the identified GO terms were upregulated in infiltrated human immune cells (Fig. 5F, G). In particular, CD4 + T cells (cl0) from the injured spinal cord showed enrichment for 24 GO terms, including several relating to cell activity (oxidative phosphorylation, proton transport, mitochondrial ATP synthesis) and immune function (antigen processing and presentation of endogenous and exogenous peptides, positive regulation of T cell proliferation). Treg cells (cl1) were enriched only for antigen processing and presentation of endogenous antigen, due to an increase in the expression of antigen presentation-related genes (e.g. HLA-B, CD74). Memory CD8 + T cells (cl4) were enriched for 9 GO terms, including response to interferon-gamma, negative regulation of leukocyte mediated cytotoxicity and antigen processing and presentation of endogenous peptide. Basophils (cl9) were enriched for 4 GO terms, all of which related to general cell functions. Finally, infiltrated neutrophils (cl10) were enriched for 14 GO terms, nine of which related to general cell function and metabolism, while other immune-associated GO terms related to an upregulation of genes involved in the cellular response to interferon gamma, antigen processing of exogenous and endogenous peptide, and T cell-mediated cytotoxicity.

Cell-to-cell interaction (CCI) analysis provided additional insights as to what was taking place at the lesion site in terms of signalling between infiltrated human immune cells. Varying levels of communication were observed within and between the main clusters (CD4 + and CD8 + T cells, and granulocytes; Additional file 1: Fig. S7C). Subsequent analysis of communication patterns [33] did not uncover evidence of different immune cell types signalling together; rather, we identified five incoming and five outgoing signalling patterns, each of which was associated with a single cell type (Additional file 1: Fig. S7D,E). Outgoing signals from memory CD8 + T cells (cl4) to other cells were predominantly associated with chemokine and interferon pathways known to recruit and/or activate other immune cells, antigen presentation for neutrophils (cl10), while basophils sent signals in relation to the regulation of cytokine production, T cell function and maintenance (Additional file 1: Fig. S7D,F). In terms of input, neutrophils mostly received signals regulating cell adhesion and myeloid cell activation, and CD4 + T cells (cl0) in relation to cytotoxicity and function of natural killer cells, as per the identified GO terms (Additional file 1: Fig. S7G), but with the identified signalling pathways (Additional file 1: Fig. S7E) known to directly regulate the recruitment, adhesion and activation state of these cells.

Together, these results provide novel insights into how infiltrated human immune cells are transcriptionally distinct to their circulating counterparts under SCI conditions, and how they are activated by and/or communicate with each other within the microenvironment of the lesion site.

#### Humanised mice recapitulate some aspects of SCI-associated immune depression syndrome in human patients

**Fig. 6:**
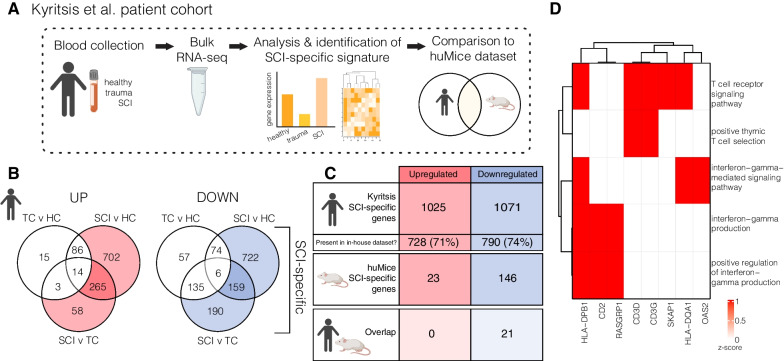
Congruence of the SCI-specific gene signature identified in huNSG-SGM3 mice with that of human patients. **A** Experimental workflow for the reference human patient dataset [2]. **B** Venn diagram showing the genes up- (left) and downregulated (right) in trauma control (TC) v healthy control (HC), spinal cord injury (SCI) v HC, and SCI v TC comparisons in the reference dataset. Coloured parts of venn diagram indicate the SCI-specific genes. **C** Table showing the number of SCI-specific genes in the human dataset from Kyritsis et al. [2] (row 1), their overall presence within the humanised mouse dataset (row 2), the number of SCI-specific (i.e. regulated) genes in the humanised mouse dataset (row 3), and the number of human SCI-specific genes that overlap with the huMice SCI-specific genes (row 4). **D** The 5 gene ontology (GO) terms linked to the 21 overlapping downregulated genes identified in C. Driver genes regulating these GO terms are shown on the *x*-axis

We lastly aimed to determine to what extent the SCI-specific gene signature, as elicited within our humanised mice, matches that of human patients. For this, we compared blood scRNAseq results from huNSG-SGM3 mice with a publicly available bulk RNA-seq dataset from human blood [1] (Fig. 6A); the latter dataset includes whole blood samples from healthy controls (HC), trauma patients with no known neurological injury (trauma controls; TC), and patients with acute SCI.

We firstly (re-)identified the SCI-specific genes that were either up- or down-regulated in response to SCI within the reference dataset (coloured parts of Venn Diagram; Fig. 6B). In doing so, we confirmed that 1025 genes were upregulated and 1071 genes downregulated following SCI compared to either the HC or TC samples; 71% of the upregulated genes and 74% of the downregulated genes were also be detected within our humanised mouse dataset (Fig. 6C). We then compared how well the ‘SCI-specific’ genes from human patients in the Kyritsis et al*.* dataset [1] matched those identified in our huNSG-SGM3 mice. Although no overlap in upregulated genes was detected, we did find a total of 21 downregulated genes that were shared between the two datasets (Fig. 6C, bottom). Further research will be needed to understand how timepoint selection and also the underdevelopment of certain human immune cell lineages in our huNSG-SGM3 mouse model affected outcomes here in terms of overlap. Nevertheless, the shared downregulated genes all linked to GO terms involved in T cell receptor signalling and interferon signalling, suggesting that these patient-specific changes are conserved and recapitulated in mice with humanised immune systems (Figs. 6D and Fig. 4).

## Discussion

Humanised mouse models provide unique opportunities to better understand the function and behaviour of human immune cells in health and disease. Here we provided a comprehensive transcriptome analysis of the human immune cell repertoire in huNSG-SGM3 mice, with an added focus as to how these cells respond to tissue injury. Using both sham-operated (trauma controls) and SCI mice, we identify a gene signature in circulating human immune cells that is specific to neurotrauma, and we also show how these cells change as they enter into the lesion site. We further provide insights into molecular underpinnings and/or transcriptional changes associated with SCI-IDS, that is, the reduced functional status of immune cells following such insult.

### scRNAseq provides insights SCI-associated immune depression syndrome

The phenomenon of SCI-IDS following neurological injury has been widely reported in both humans [2] and mice [3, 35]. SCI-IDS is thought to result from autonomic dysfunction and known to reduce both B and T cell function [3, 36], cell types that were well represented in our huNSG-SGM3 model. A detailed molecular characterisation of the SCI-IDS phenomenon has, however, been lacking. In the present study, we were able to identify a specific suite of genes that was downregulated within circulating human immune cells after SCI compared to sham and/or naïve samples. The SCI-specific response in our humanised mouse model was hallmarked by an overall decrease in inflammatory functions, including Type I and II interferon signalling, antigen processing and presentation. These findings are consistent with reported functional changes in immune status in non-humanised rodent models of SCI [36, 37].

Comparison of scRNAseq data from our humanised mouse model with bulk RNAseq data from human patients [1] revealed a consensus set of 21 genes that were downregulated across both datasets. Further work is needed to better understand the impact of divergent WBC profiles and also timepoint selection on the limited overlap in regulated genes. Indeed, transcriptome deconvolution approaches showed neutrophils and monocytes represented > 70% of sequenced immune cell types in the Kyritsis et al. dataset [1], while these myeloid lineages remained underdeveloped in the huNSG-SGM3 mouse model. Comparisons with other humanised mouse models that may better support the development of human innate immune cells [8], and also a more acute timepoint for cell isolation in experimental studies (1 as opposed to 7dpi) could help to reconcile these differences. Nevertheless, shared downregulated genes were all strongly linked to T cell and interferon signalling, providing evidence that at least some functional aspects of SCI-IDS are shared between the huNSG-SGM3 model and human patients, in addition to giving insights into the molecular underpinnings of this immunosuppressive phenotype. Future studies could investigate whether interferon therapy can be used to overcome and/or treat acute SCI-IDS.

### Engraftment of human immune cells worsens SCI recovery of NSG-SGM3 mice

We found that non-engrafted NSG-SGM3 mice recovered significantly better from contusive SCI than immunocompetent C57BL/6J mice, and also that this advantage was lost with the introduction of human immune cells. These findings are consistent with other independent reports using immunocompromised and/or humanised mouse strains [9, 38, 39]. We sought to better understand therefore how the introduction and presence of human immune cells may have altered the lesion microenvironment, and ultimately recovery. Despite the observed SCI-IDS, we found that infiltrated human immune cells were significantly enriched for GO terms relating to cellular metabolism and inflammation, signifying their activation as they migrate from the blood into the injured spinal cord. To what extent these transcriptional changes match what occurs in human patients is difficult to discern, but evidence of significant inflammation and immune cell activity at the site of SCI in human patients does exist [4, 5].

The present findings lay a foundation for future studies to probe the relationship between human immune cell presence and the recovery of NSG-SGM3 from SCI. Both neutrophils and CD8 + T cells are obvious candidates for follow-up, and cell depletion and/or gene targeting studies could delineate the extent to which the identified immune signalling pathways influence the functional and/or histopathological outcomes. That said, it should be recognised that immune cell presence and/or states are influenced by cues from within the local microenvironment [40, 41], and the extent to which the observed transcriptional changes (or pathways) are influenced by the underdevelopment and/or limited presence of human innate immune cells remains to be determined. On a separate note, it would be of interest for any future studies to also profile the murine immune cell lineages that remain in the complex genetic background of NSG and/or NSG-SGM3 strains, including microglia, to understand their interactions with human immune cells at the site of SCI.

### Limitations and concluding remarks

To the best of our knowledge, this is the first study conducting an in-depth profiling of the human immune cell repertoire in huNSG-SGM3 mice, and how these cells change and/or respond to (neuro-)trauma. As alluded to earlier, the NSG-SGM3 strain was developed as a “next-generation” humanised mouse model to augment development of the human myeloid compartment [11]. However, considerable defects in innate human immune cell development were still observed in our study, and similar to NSG mice [8, 12], grafted cells mostly gave rise to lymphoid lineages. Studying human myeloid cell biology thus remains challenging and this shortcoming needs to be addressed going forward in order for mice with humanised immune systems to reach their full translational potential and promise. Martinov and colleagues [8] already extensively discussed ongoing developments in this space, also reviewing the individual strengths and limitations of current humanised mouse models, along with the challenges that remain around (the provision of) niches that can better support various human immune lineages, lymphoid organ development/functionality, and the interactions between human immune effector cells and mouse tissues more broadly. In the context of neurotrauma, where innate immunity dominates during the (sub-) acute phase, exogenous administration of CSF-1 [42] may provide one avenue to better support human myelo-monocytic cell differentiation [8]; MISTRG mice could offer a genetic alternative here, as they not only express GM-CSF/CSF-2 (like the NSG-SGM3 mice used here) but also CSF-1 [43]. Improved development of human monocytes and macrophages may come at a cost, however, as xenogeneic haemophagocytosis (clearance of mouse red blood cells by human phagocytic cells) can cause lethal anaemia under such conditions [43]. More work is needed therefore to promote phagocytic tolerance in humanised mouse models and/or to stimulate human red blood cell formation from the graft.

Interestingly, huNSG-SGM3 mice can reportedly also suffer from macrophage activation syndrome and associated anaemia [44], along with bone marrow hypoplasia, macrophage-derived hypercytokinema and the infiltration of human T cells and macrophages into the lung and liver [45]. Most of these pathologies were, however, observed from 18 weeks post-engraftment, and huNSG-SGM3 mice in our study were all sacrificed by 16 weeks; that is, prior to the onset of these symptoms [45]. Our huNSG-SGM3 mice indeed showed normal, near-complete recovery of body weight post-SCI and no deterioration thereof over time (Additional file 1: Fig. S8H), although some immune infiltrate was observed in the livers of our huNSG-SGM3 mice (Additional file 1: Fig. S8A–F). Whether differentiation of human myelo-monocytic cells into macrophages occurs only relatively late in humanised NSG-SGM3 mice and/or under specific conditions remains to be determined. Regardless, and in contrast to observations made by others [45], we found no evidence that possible graft-versus-host disease (GVHD) in these mice was influenced and/or exacerbated by a neurotraumatic event like SCI Additional file 1: Fig. S8G).

Despite the above limitations, our results show that aspects of SCI-IDS can be modelled and/or are recapitulated in humanised mice, providing avenues for further research into this adverse phenomenon and how it may be mitigated. As stated, we do not believe that the possible presence of low-grade GVHD confounded our results, as it was equally present in all mice regardless of the experimental condition and/or timepoint (Additional file 1: Fig. S8). We also identified a gene signature that is induced specifically upon the entry of human immune cells into the injured spinal cord, the functional significance of which can now be probed further. Although a relatively large number of cells (pooled from different animals) was profiled in our scRNAseq experiments, we do acknowledge that the lack and/or limited number of technical replicates does carry a risk of overgeneralisation; independent replication and future validation studies of interesting targets will therefore strengthen the conclusions drawn from DEG and CCI analyses. That said, the independent flow cytometric validation of cell types, and also the cross-validation with human data, already provides a reasonable level of confidence in the inferences made from the specific cases presented here. A final limitation to be considered is the low number of human immune cells recovered from the spinal cord lesion site itself. Innovations in mouse models that better recapitulate human immunity and have a full and/or more complete spectrum of myeloid cells (which dominate spinal cord lesion sites at all injury phases [4, 5]), may partially overcome this problem in the future. Varying the timing of collection may also improve yield, with human immune cell presence seemingly appearing more robust (and diverse) at later timepoints [9, 10]; however, the disadvantage of this approach would be that it also changes the temporal phase of lesion site development. Pooling multiple samples of injured spinal cord may therefore provide the most practical solution at present to address the imbalance in human immune cell yield from the CNS) relative to the blood. That said, it will always be challenging to obtain an equal number of human immune cell transcriptomes across both compartments, as the number of cells that is recruited to (and thus recoverable from) inflamed sites is typically only a fraction of those circulating. Downsampling the latter would inevitably come with information loss, and careful consideration should be given therefore as to whether any numerical differences in cellular input influence clustering results.

Overall, we conclude that huNSG-SGM3 mice appear well suited to probe the role of human neutrophils and various lymphoid populations in both SCI-IDS and SCI pathology [5, 38, 46, 47], with the caveat that the composition of the overall immune infiltrate (or changes therein) may affect functional outcomes [6, 48, 49].

### Supplementary Information

**Additional file 1: Figure S1.** Blood profile of non-humanised NSG-SGM3 mice. A) NSG-SGM3 mice without human immune reconstitution are lymphopenic and have fewer white blood cells (WBCs) compared to wild type C57BL6/J mice. Neu: neutrophils, Lym: lymphocytes, Mon: monocytes, Eos: eosinophils, Bas: basophils. Data points represent blood counts from individual animals, with the mean and standard error of the mean (SEM) also indicated. Two-way ANOVA with Sidak’s post hoc; ****, *p*<0.0001. **Figure S2.** Blood profile of humanised NSG-SGM3 mice. A) Representative flow cytometry plots and gating strategy for human and mouse immune cell populations. B and C) Composition of human (B) and mouse (C) CD45+ cells in the blood at 16 weeks post-engraftment (42 days post-injury). Data points in (B) and (C) represent blood counts from individual animals, with the mean and standard error of the mean (SEM) also indicated. Statistical analysis was performed using two-way ANOVA with Tukey’s post-hoc (B) or unpaired student’s t-test (C); *n*=7/group;); *, *p*<0.05; ***, *p*<0.001; ****, *p*<0.0001. **Figure S3.** Gating strategy for the isolation of human immune cells. A) Representative FACS plots for blood (left) and spinal cord (right) and gating strategy used for the sorting of human immune cells (i.e., huCD45+; orange gate) from blood and spinal cord samples. **Figure S4.** Evidence for immunoglobulin (Ig) class switching, granzyme and perforin expression by human lymphocytes. A to C) Human IgG (huIgG; magenta) staining, or the appropriate lack thereof, in representative spleen sections from C57BL6/J (A), NSG-SGM3 (B) and humanised (hu) NSG-SGM3 mice (C); Hoechst+ cell nuclei are shown in cyan. Scale bar is 100 μm. D and E) UMAP plots showing gene expression levels of select immunoglobulin genes (D) and granzyme/perforin genes (E) with cluster annotation within the huMice single-cell RNAseq atlas. Colour represents z-score expression level. **Figure S5.** Clustering and/or colocalisation of human immune cells relative to the experimental condition. A to C) MetaCell networks for circulating leukocytes, split into major cell types and subsets (see cluster numbers), for T cells (A), B cells (B), and granulocytes (C). Cells are colour-coded by experimental condition, i.e., naïve (yellow), sham (light blue) and spinal cord injury (SCI; dark blue) to visualise their (co-) location within the MetaCell network diagram. **Figure S6.** Non-neurological trauma mostly increases inflammation and interactions between circulating human immune cells whilst SCI decreases this. A) Number of differentially expressed genes (DEGs) (i.e., markers; left) and corresponding significant gene ontology (GO) terms (right), as identified for each cluster in response to tissue injury (i.e., between sham and naïve; coloured part of Venn Diagram). B) Top regulated GO terms relating to DEGs between sham versus (v) naïve conditions, for each classified cell cluster. C) GO terms regulated in the same direction in both ‘sham v naïve’ and ‘SCI v naïve’ comparisons (i.e. reflecting trauma-associated rather than SCI-specific changes in GO terms; coloured part of Venn Diagram). For GO plots in B  and C, blue colour represents the log10(adjusted *p*-value) for downregulated genes/terms while red colour would represent –log10(adjusted *p*-value) for upregulated genes/terms; dot size represents ratio of genes contributing to the GO term. (D) Number (top) and strength (bottom) of cell-to-cell interaction (CCI) events predicted to occur in blood samples from naïve, sham and SCI mice, based on known ligand-receptor (LR) pairs. (E) Network diagrams showing differential communication between pairs of cell types (clusters) under the specified conditions. Red lines indicate greater connectivity in the first-named sample compared to the second; blue lines indicate a downregulation, as in a greater connectivity in the second-named sample compared to the first. Only the top 20% of connections are shown, and the relative number and/or strength of predicted CCIs is indicated by the line weight. (F) Top 6 enriched GO terms associated with CCI events predicted to be upregulated for comparisons shown in E (bottom plots, interaction strength); LR pairs that were filtered to remove pairs enriched in the reference (i.e. blue) condition were removed prior to GO analysis. **Figure S7.** Transcriptome comparison and communication between infiltrated human immune cells. A) Violin plots showing log expression of the top regulated genes in huCD45+ cells isolated from the spinal cord (green) and blood (pink). B) Top gene ontology (GO) terms enriched in human immune cells isolated from the injured spinal cord compared to those in the circulation (i.e., cord v blood) across whole samples. Red colour represents –log10(adjusted *p*-value) for upregulated genes/terms; dot size represents ratio of genes contributing to the GO term. C) Predicted cell-to-cell interactions (CCIs) between identified human immune cell types in the injured spinal cord, based on known interactions between ligands (L; left) and receptors (R; right). Coloured segments around each half of the plot represent cell clusters, grouped into main immune cell types as per the annotation in the grey bars/segments. Connecting line widths indicate the number of predicted ligand-receptor (LR) events between the connected cell types; line colours indicate sender cell identity. D) Outgoing communication patterns for named cell clusters (left). Unique signalling patterns (P) were predicted for each, with a total of five outgoing patterns detected, respectively (middle; P1-P5). Signalling pathways (encompassing one or more LR pairs) associated with each pattern are also shown (right). E) As for D, but now showing unique incoming communication patterns (i.e., signals received) for each cell cluster. F) Top 5 significant GO terms associated with the outgoing signalling patterns shown in D. Ligands predicted to transmit the outgoing signals were used as input for GO enrichment analysis; no significant hits were found for P4 and P5. G) As for F, but using receptors for incoming signals (highlighted in E) instead for GO enrichment analysis. **Figure S8.** Histological evidence of graft-versus-host disease in humanised NSG-SGM3 mice. A to F) Representative images of H&E-stained livers showing perivascular immune infiltrate in humanised (hu) NSG-SGM3 mice (denoted by arrows), characteristic of graft-versus-host disease (GVHD). Scale bar is 100μm. G) GVHD scores for livers of naïve, sham and SCI (1, 7 and 42 days post-injury; dpi); non-humanised NSG-SGM3 mice were included as an additional control. A score of ‘0’ (no visible pathology) is visually represented in (A) and a score of ‘3’ (sporadic perivascular infiltration with some additional spread into the parenchyma) in (B-F). H) Bodyweight of NSG-SGM3 and huNSG-SGM3 mice following SCI (normalised to pre-surgery weight). Statistical analyses were performed using one-way ANOVA with Tukey’s post hoc (G), or a mixed effects model with Sidak’s multiple comparisons test (H); *, *p*<0.05; **, *p*<0.01; ***, *p*<0.001.

## Data Availability

Raw and processed sequencing data, generated as part of this study, have been deposited to the Gene Expression Omnibus repository (https://www.ncbi.nlm.nih.gov/geo/) and are available publicly under the accession number GSE236293. All other experimental data can be made available upon request and/or found at the ODC-SCI repository (odc-sci.org). Code to reproduce scRNAseq figures presented in this paper is available at our dedicated GitHub site for maPping cellulaR ECosystems in SpInal cOrd iNjury (PRECISION): https://github.com/BiomedicalMachineLearning/PRECISION.

## Abbreviations

BMS: Basso mouse scale
CCI: Cell-to-cell interaction
cl: Cluster
CNS: Central nervous system
DEG: Differentially expressed gene
DPBS: Dulbecco’s phosphate-buffered saline
dpi: Days post-injury
EDTA: Ethylenediminetetraacetic acid
FACS: Fluorescence activated cell sorting
GO: Gene ontology
GVHD: Graft-versus-host disease
HC: Healthy control
HSPC: Haematopoietic stem/progenitor cells
hu: Human
huCD14: Human CD14
huCD11b: Human CD11b
huCD45: Human CD45
huNSG: Humanised NSG
huNSG-SGM3: Human NSG-SGM3
huWBC: Human white blood cells
IP: Intra-peritoneal
MAD: Median absolute deviation
ms: Mouse
msCD11b: Mouse CD11b
msCD45: Mouse CD45
NOD: Nonobese diabetic
NSG-SGM3: NOD-SCID-gamma, SCF-KIT ligand, GM-CSF/colony stimulating factor 2 and IL-3
RBC: Red blood cell
RT: Room temperature
SC: Sub-cutaneous
SCI: Spinal cord injury
SCID: Severe combined immunodeficient
SCI-IDS: Spinal cord injury-induced systemic immune depression syndrome
scRNAseq: Single cell RNA-sequencing
SEM: Standard error of the mean
SGM3: SCF-KIT ligand, GM-CSF/colony stimulating factor 2 and IL-3
TC: Trauma control
WBC: White blood cell
