# Supplementary material for: Single-cell RNA sequencing reveals peripheral blood leukocyte responses to spinal cord injury in mice with humanised immune systems
Source: J Neuroinflammation. 2024 Mar 1;21:63. doi: 10.1186/s12974-024-03048-0 (PMC10908016; doi:10.1186/s12974-024-03048-0)
Supplement: Supplementary file 1 — Additional file 1: Figure S1. Blood profile of non-humanised NSG-SGM3 mice. A) NSG-SGM3 mice without human immune reconstitution are lymphopenic and have fewer white blood cells (WBCs) compared to wild type C57BL6/J mice. Neu: neutrophils, Lym: lymphocytes, Mon: monocytes, Eos: eosinophils, Bas: basophils. Data points represent blood counts from individual animals, with the mean and standard error of the mean (SEM) also indicated. Two-way ANOVA with Sidak’s post hoc; ****, p<0.0001. Figure S2. Blood profile of humanised NSG-SGM3 mice. A) Representative flow cytometry plots and gating strategy for human and mouse immune cell populations. B and C) Composition of human (B) and mouse (C) CD45+ cells in the blood at 16 weeks post-engraftment (42 days post-injury). Data points in (B) and (C) represent blood counts from individual animals, with the mean and standard error of the mean (SEM) also indicated. Statistical analysis was performed using two-way ANOVA with Tukey’s post-hoc (B) or unpaired student’s t-test (C); n=7/group;); *, p<0.05; ***, p<0.001; ****, p<0.0001. Figure S3. Gating strategy for the isolation of human immune cells. A) Representative FACS plots for blood (left) and spinal cord (right) and gating strategy used for the sorting of human immune cells (i.e., huCD45+; orange gate) from blood and spinal cord samples. Figure S4. Evidence for immunoglobulin (Ig) class switching, granzyme and perforin expression by human lymphocytes. A to C) Human IgG (huIgG; magenta) staining, or the appropriate lack thereof, in representative spleen sections from C57BL6/J (A), NSG-SGM3 (B) and humanised (hu) NSG-SGM3 mice (C); Hoechst+ cell nuclei are shown in cyan. Scale bar is 100 μm. D and E) UMAP plots showing gene expression levels of select immunoglobulin genes (D) and granzyme/perforin genes (E) with cluster annotation within the huMice single-cell RNAseq atlas. Colour represents z-score expression level. Figure S5. Clustering and/or colocalisation of human immune cell [file 12974_2024_3048_MOESM1_ESM.pdf]

Additional material

Figure S1

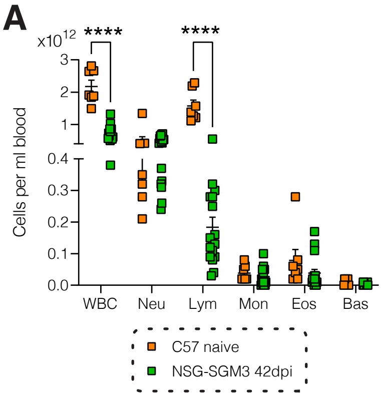

Figure S1. Blood profile of non-humanised NSG-SGM3 mice. A) NSG-SGM3 mice without human immune reconstitution are lymphopenic and have fewer white blood cells (WBCs) compared to wild type C57BL6/J mice. Neu: neutrophils, Lym: lymphocytes, Mon: monocytes, Eos: eosinophils, Bas: basophils. Data points represent blood counts from individual animals, with the mean and standard error of the mean (SEM) also indicated. Two-way ANOVA with Sidak's post hoc; ****, $p<0.0001$.

Figure S2

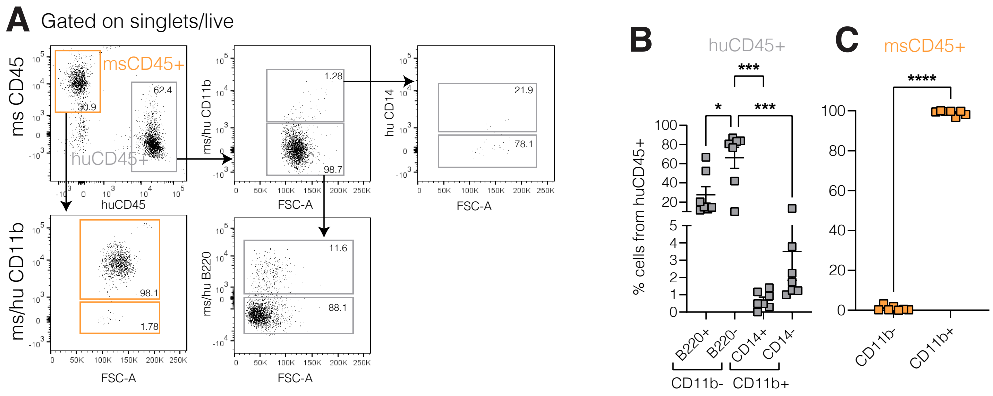

Figure S2. Blood profile of humanised NSG-SGM3 mice. A) Representative flow cytometry plots and gating strategy for human and mouse immune cell populations. B and C) Composition of human (B) and mouse (C) CD45+ cells in the blood at 16 weeks post-engraftment (42 days post-injury). Data points in (B) and (C) represent blood counts from individual animals, with the mean and standard error of the mean (SEM) also indicated. Statistical analysis was performed using two-way ANOVA with Tukey's post-hoc (B) or unpaired student's t-test (C); *n*=7/group;); *, $p<0.05$; ***, $p<0.001$; ****, $p<0.0001$.

Figure S3

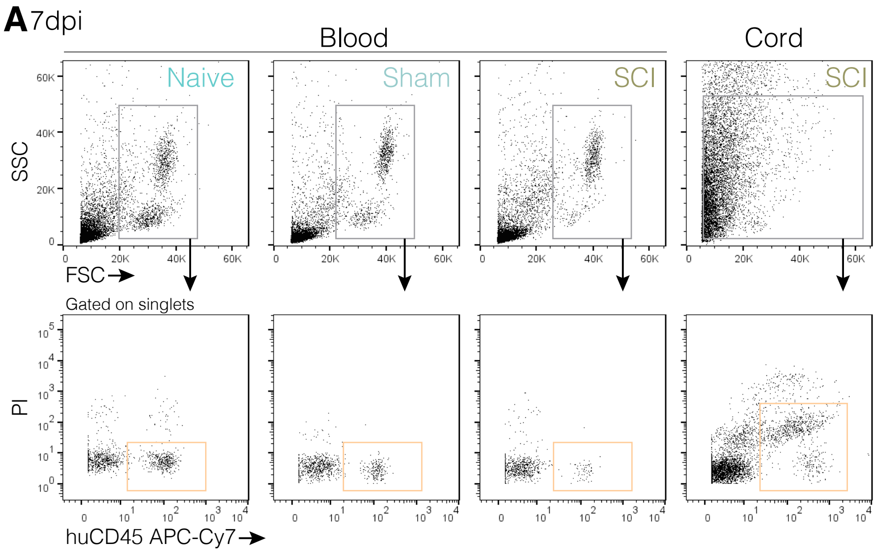

Figure S3. Gating strategy for the isolation of human immune cells. A) Representative FACS plots for blood (left) and spinal cord (right) and gating strategy used for the sorting of human immune cells (i.e., huCD45+; orange gate) from blood and spinal cord samples.

Figure S4

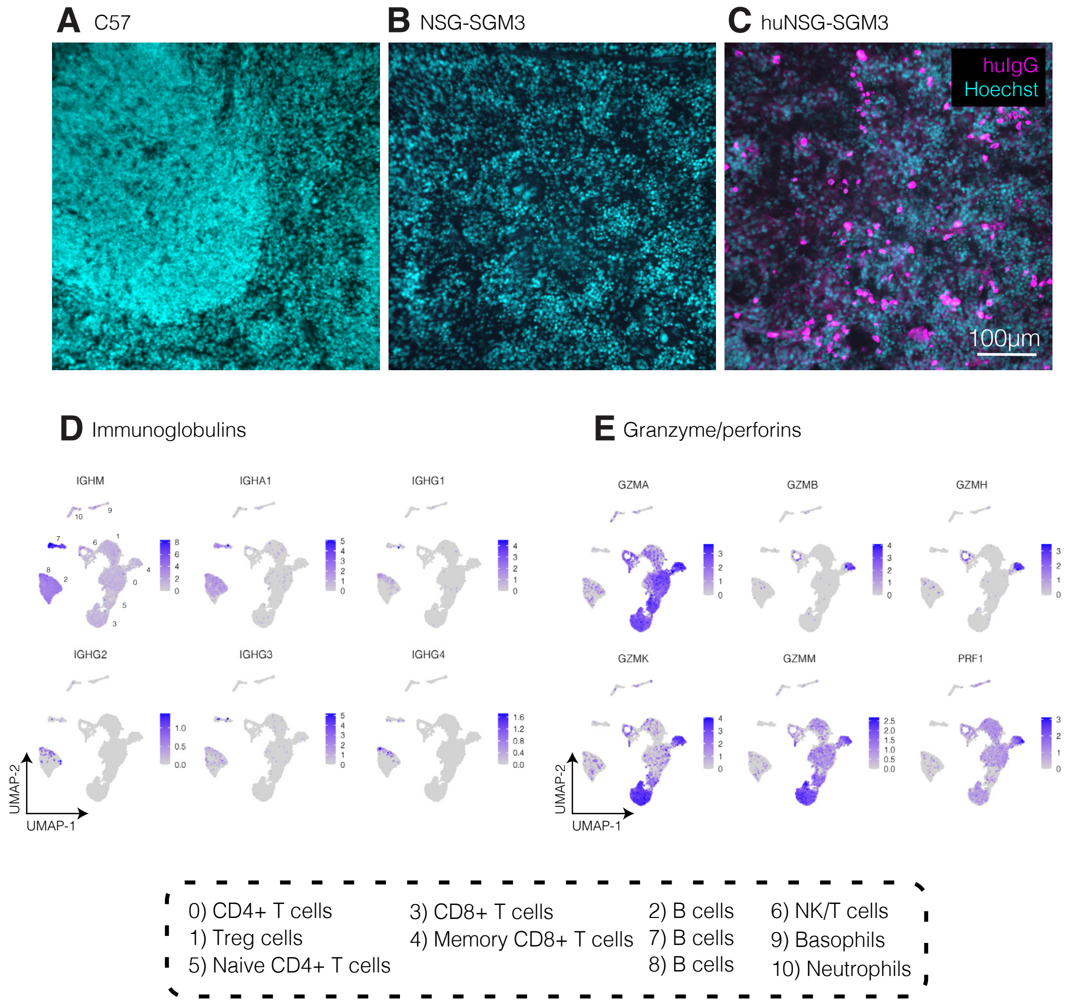

Figure S4. Evidence for immunoglobulin (Ig) class switching, granzyme and perforin expression by human lymphocytes. A to C) Human IgG (huIgG; magenta) staining, or the appropriate lack thereof, in representative spleen sections from C57BL6/J (A), NSG-SGM3 (B) and humanised (hu) NSG-SGM3 mice (C); Hoechst+ cell nuclei are shown in cyan. Scale bar is 100µm. D and E) UMAP plots showing gene expression levels of select immunoglobulin genes (D) and granzyme/perforin genes (E) with cluster annotation within the huMice single-cell RNAseq atlas. Colour represents z-score expression level.

Figure S5

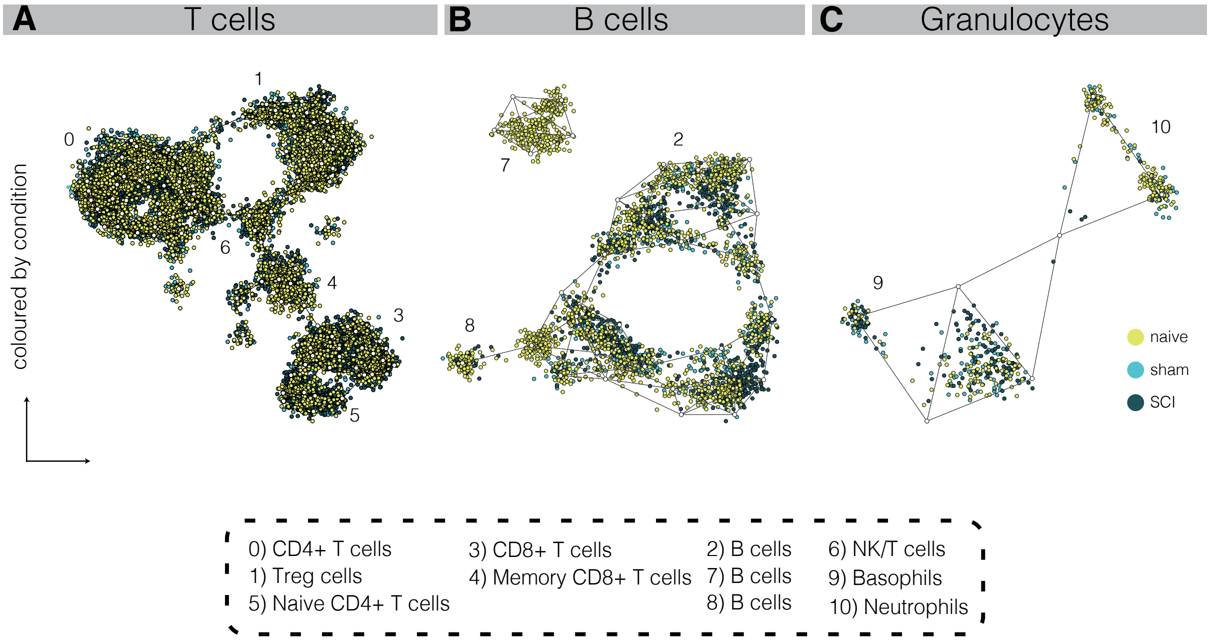

Figure S5. Clustering and/or colocalisation of human immune cells relative to the experimental condition. A to C) MetaCell networks for circulating leukocytes, split into major cell types and subsets (see cluster numbers), for T cells (A), B cells (B), and granulocytes (C). Cells are colour-coded by experimental condition, i.e., naïve (yellow), sham (light blue) and spinal cord injury (SCI; dark blue) to visualise their (co-) location within the MetaCell network diagram.

Figure S6

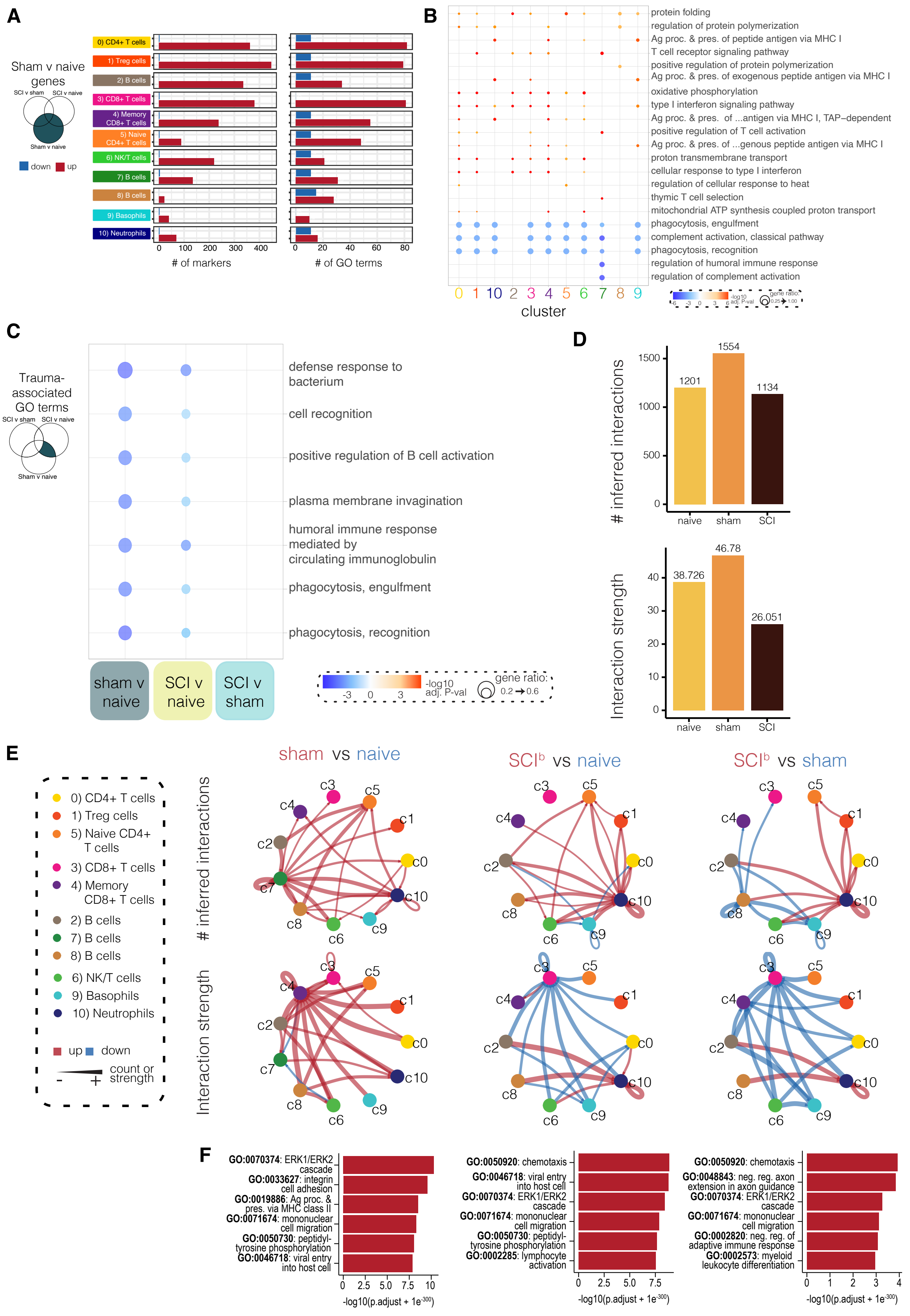

A
Sham v naive genes
SCI v sham
SCI v naive
Sham v naive
down
up
0) CD4+ T cells
1) Treg cells
2) B cells
3) CD8+ T cells
4) Memory CD8+ T cells
5) Naive CD4+ T cells
6) NK/T cells
7) B cells
8) B cells
9) Basophils
10) Neutrophils
# of markers
# of GO terms
B
protein folding
regulation of protein polymerization
Ag proc. & pres. of peptide antigen via MHC I
T cell receptor signaling pathway
positive regulation of protein polymerization
Ag proc. & pres. of exogenous peptide antigen via MHC I
oxidative phosphorylation
type I interferon signaling pathway
Ag proc. & pres. of ...antigen via MHC I, TAP−dependent
positive regulation of T cell activation
Ag proc. & pres. of ...genous peptide antigen via MHC I
proton transmembrane transport
cellular response to type I interferon
regulation of cellular response to heat
thymic T cell selection
mitochondrial ATP synthesis coupled proton transport
phagocytosis, engulfment
complement activation, classical pathway
phagocytosis, recognition
regulation of humoral immune response
regulation of complement activation
cluster
C
Trauma-associated GO terms
defense response to bacterium
cell recognition
positive regulation of B cell activation
plasma membrane invagination
humoral immune response mediated by circulating immunoglobulin
phagocytosis, engulfment
phagocytosis, recognition
sham v naive
SCI v naive
SCI v sham
-log10 adj. P-val
gene ratio:
D
inferred interactions
1201
1554
1134
Interaction strength
38.726
46.78
26.051
naive
sham
SCI
E
sham vs naive
SCI^b vs naive
SCI^b vs sham
0) CD4+ T cells
1) Treg cells
5) Naive CD4+ T cells
3) CD8+ T cells
4) Memory CD8+ T cells
2) B cells
7) B cells
8) B cells
6) NK/T cells
9) Basophils
10) Neutrophils
up
down
count or strength
inferred interactions
Interaction strength
F
GO:0070374: ERK1/ERK2 cascade
GO:0033627: integrin cell adhesion
GO:0019886: Ag proc. & pres. via MHC class II
GO:0071674: mononuclear cell migration
GO:0050730: peptidyl-tyrosine phosphorylation
GO:0046718: viral entry into host cell
GO:0050920: chemotaxis
GO:0002285: lymphocyte activation
GO:0048843: neg. reg. axon extension in axon guidance
GO:0002820: neg. reg. of adaptive immune response
GO:0002573: myeloid leukocyte differentiation
-log10(p.adjust + 1e-300)

(See figure on previous page)

Figure S6. Non-neurological trauma mostly increases inflammation and interactions between circulating human immune cells whilst SCI decreases this. A) Number of differentially expressed genes (DEGs) (i.e., markers; left) and corresponding significant gene ontology (GO) terms (right), as identified for each cluster in response to tissue injury (i.e., between sham and naïve; coloured part of Venn Diagram). B) Top regulated GO terms relating to DEGs between sham versus (v) naïve conditions, for each classified cell cluster. C) GO terms regulated in the same direction in both 'sham v naïve' and 'SCI v naïve' comparisons (i.e. reflecting trauma-associated rather than SCI-specific changes in GO terms; coloured part of Venn Diagram). For GO plots in B and C, blue colour represents the log10(adjusted *p*-value) for downregulated genes/terms while red colour would represent –log10(adjusted *p*-value) for upregulated genes/terms; dot size represents ratio of genes contributing to the GO term. (D) Number (top) and strength (bottom) of cell-to-cell interaction (CCI) events predicted to occur in blood samples from naïve, sham and SCI mice, based on known ligand-receptor (LR) pairs. (E) Network diagrams showing differentialcommunication between pairs of cell types (clusters) under the specified conditions. Red lines indicate greater connectivity in the first-named sample compared to the second; blue lines indicate a downregulation, as in a greater connectivity in the second-named sample compared to the first. Only the top 20% of connections are shown, and the relative number and/or strength of predicted CCIs is indicated by the line weight. (F) Top 6 enriched GO terms associated with CCI events predicted to be upregulated for comparisons shown in E (bottom plots, interaction strength); LR pairs that werewere filtered to remove pairs enriched in the reference (i.e. blue) condition were removed prior to GO analysis

Figure S7

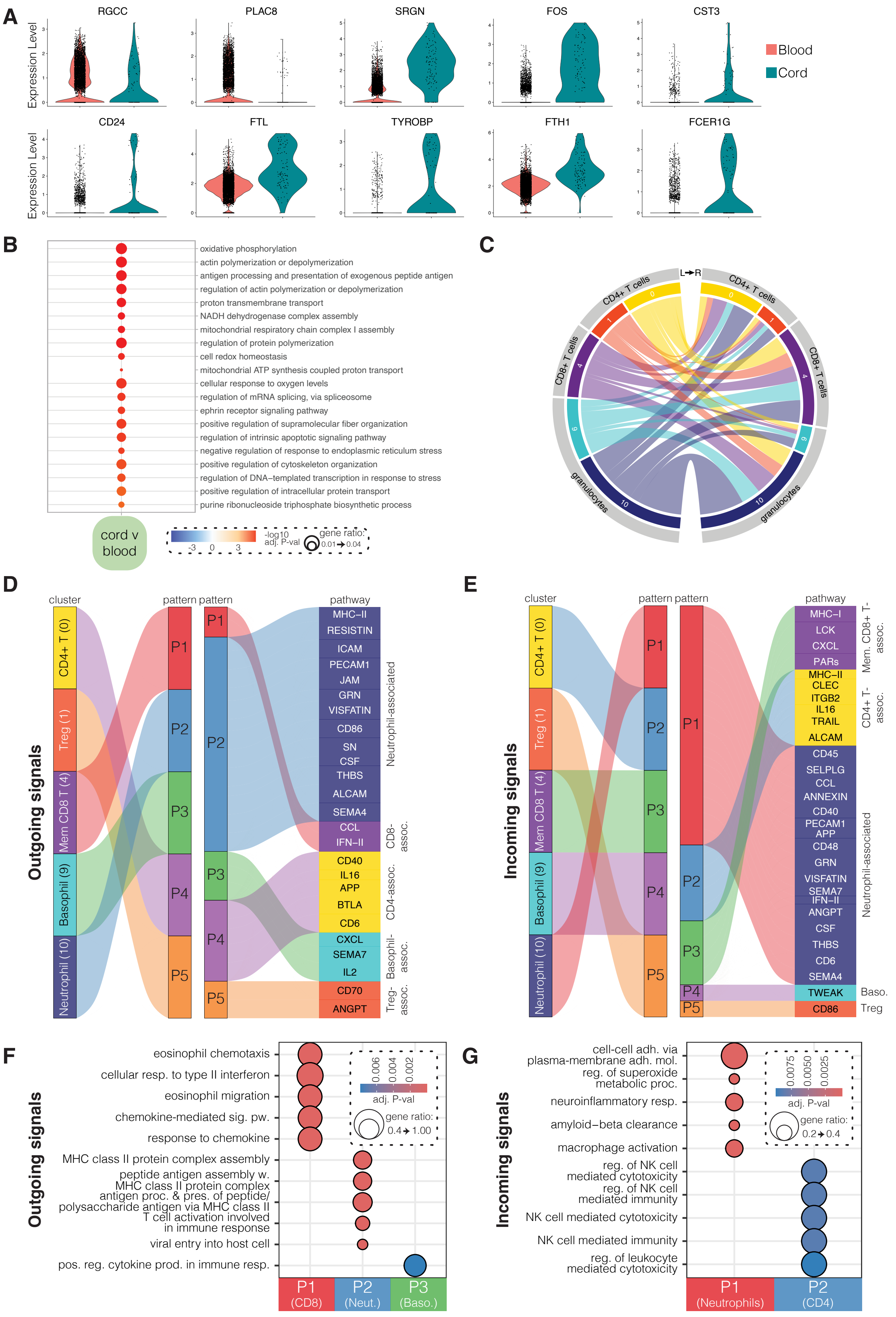

A
RGCC
PLAC8
SRGN
FOS
CST3
CD24
FTL
TYROBP
FTH1
FCER1G
Expression Level
Blood
Cord
B
oxidative phosphorylation
actin polymerization or depolymerization
antigen processing and presentation of exogenous peptide antigen
regulation of actin polymerization or depolymerization
proton transmembrane transport
NADH dehydrogenase complex assembly
mitochondrial respiratory chain complex I assembly
regulation of protein polymerization
cell redox homeostasis
mitochondrial ATP synthesis coupled proton transport
cellular response to oxygen levels
regulation of mRNA splicing, via spliceosome
ephrin receptor signaling pathway
positive regulation of supramolecular fiber organization
regulation of intrinsic apoptotic signaling pathway
negative regulation of response to endoplasmic reticulum stress
positive regulation of cytoskeleton organization
regulation of DNA–templated transcription in response to stress
positive regulation of intracellular protein transport
purine ribonucleoside triphosphate biosynthetic process
cord v blood
-log10 adj. P-val
gene ratio: 0.01→0.04
C
L→R
CD4+ T cells
CD8+ T cells
granulocytes
D
cluster
pattern
pathway
CD4+ T (0)
Treg (1)
Mem CD8 T (4)
Basophil (9)
Neutrophil (10)
P1
P2
P3
P4
P5
MHC–II
RESISTIN
ICAM
PECAM1
JAM
GRN
VISFATIN
CD86
SN
CSF
THBS
ALCAM
SEMA4
CCL
IFN–II
CD40
IL16
APP
BTLA
CD6
CXCL
SEMA7
IL2
CD70
ANGPT
Neutrophil-associated
CD8-assoc.
CD4-assoc.
Basophil-assoc.
Treg-assoc.
Outgoing signals
E
MHC–I
LCK
CXCL
PARs
MHC–II
CLEC
ITGB2
IL16
TRAIL
ALCAM
CD45
SELPLG
CCL
ANNEXIN
CD40
PECAM1
APP
CD48
GRN
VISFATIN
SEMA7
IFN–II
ANGPT
CSF
THBS
CD6
SEMA4
TWEAK
CD86
Mem. CD8+ T-assoc.
CD4+ T-assoc.
Neutrophil-associated
Baso.
Treg
Incoming signals
F
eosinophil chemotaxis
cellular resp. to type II interferon
eosinophil migration
chemokine-mediated sig. pw.
response to chemokine
MHC class II protein complex assembly
peptide antigen assembly w. MHC class II protein complex
antigen proc. & pres. of peptide/ polysaccharide antigen via MHC class II
T cell activation involved in immune response
viral entry into host cell
pos. reg. cytokine prod. in immune resp.
adj. P-val
gene ratio: 0.4→1.00
P1 (CD8)
P2 (Neut.)
P3 (Baso.)
G
cell-cell adh. via plasma-membrane adh. mol.
reg. of superoxide metabolic proc.
neuroinflammatory resp.
amyloid–beta clearance
macrophage activation
reg. of NK cell mediated cytotoxicity
reg. of NK cell mediated immunity
NK cell mediated cytotoxicity
NK cell mediated immunity
reg. of leukocyte mediated cytotoxicity
adj. P-val
gene ratio: 0.2→0.4
P1 (Neutrophils)
P2 (CD4)

(See figure on previous page)

Figure S7. Transcriptome comparison and communication between infiltrated human immune cells. A) Violin plots showing log expression of the top regulated genes in huCD45+ cells isolated from the spinal cord (green) and blood (pink). B) Top gene ontology (GO) terms enriched in human immune cells isolated from the injured spinal cord compared to those in the circulation (i.e., cord v blood) across whole samples. Red colour represents –log10(adjusted *p*-value) for upregulated genes/terms; dot size represents ratio of genes contributing to the GO term. C) Predicted cell-to-cell interactions (CCIs) between identified human immune cell types in the injured spinal cord, based on known interactions between ligands (L; left) and receptors (R; right). Coloured segments around each half of the plot represent cell clusters, grouped into main immune cell types as per the annotation in the grey bars/segments. Connecting line widths indicate the number of predicted ligand-receptor (LR) events between the connected cell types; line colours indicate sender cell identity. D) Outgoing communication patterns for named cell clusters (left). Unique signalling patterns (P) were predicted for each, with a total of five outgoing patterns detected, respectively (middle; P1-P5). Signalling pathways (encompassing one or more LR pairs) associated with each pattern are also shown (right). E) As for D, but now showing unique incoming communication patterns (i.e., signals received) for each cell cluster. F) Top 5 significant GO terms associated with the outgoing signalling patterns shown in D. Ligands predicted to transmit the outgoing signals were were used as input for GO enrichment analysis; no significant hits were found for P4 and P5. G) As for F, but using receptors for incoming signals (highlighted in E) instead for GO enrichment analysis.

Figure S8

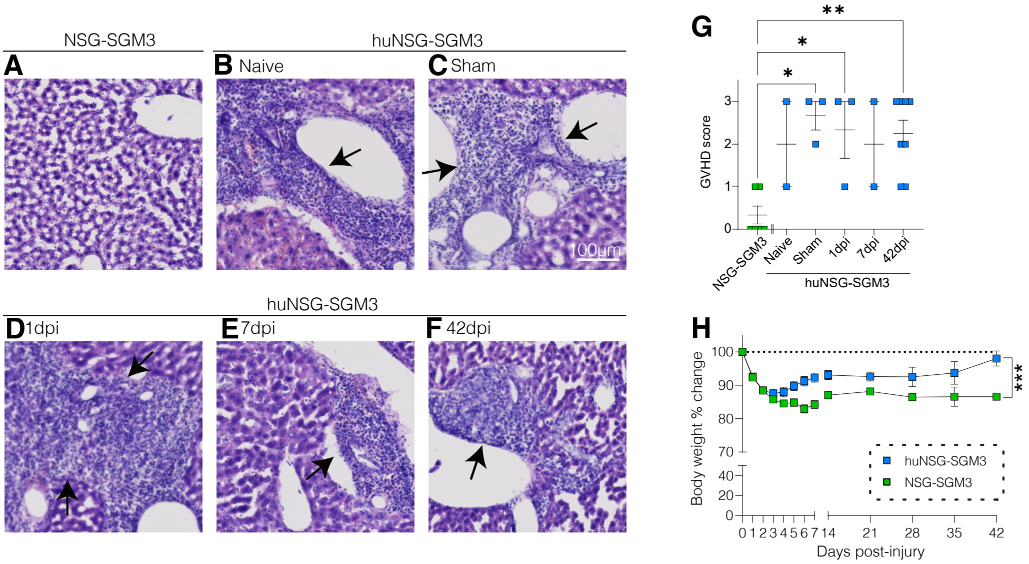

Figure S8. Histological evidence of graft-versus-host disease in humanised NSG-SGM3 mice. A to F) Representative images of H&E-stained livers showing perivascular immune infiltrate in humanized (hu) NSG-SGM3 mice (denoted by arrows), characteristic of graft-versus-host disease (GVHD). Scale bar is 100µm. G) GVHD scores for livers of naïve, sham and SCI (1, 7 and 42 days post-injury; dpi); non-humanised NSG-SGM3 mice were included as an additional control. A score of '0' (no visible pathology) is visually represented in (A) and a score of '3' (sporadic perivascular infiltration with some additional spread into the parenchyma) in (B-F). H) Bodyweight of NSG-SGM3 and huNSG-SGM3 mice following SCI (normalised to pre-surgery weight). Statistical analyses were performed using one-way ANOVA with Tukey's post hoc (G), or a mixed effects model with Sidak's multiple comparisons test (H); *, p<0.05; **, p<0.01; ***, p<0.001.
